# Integrative Taxonomic, Ecological and Genotyping Study of Charophyte Populations from the Egyptian Western-Desert Oases and Sinai Peninsula

**DOI:** 10.3390/plants10061157

**Published:** 2021-06-07

**Authors:** Abdullah A. Saber, Andrey A. Gontcharov, Arthur Yu. Nikulin, Vyacheslav Yu. Nikulin, Walaa A. Rayan, Marco Cantonati

**Affiliations:** 1 Botany Department, Faculty of Science, Ain Shams University, Abbassia Square, Cairo 11566, Egypt; abdullah_elattar@sci.asu.edu.eg (A.A.S.); walaagenetics@yahoo.com (W.A.R.); 2 Federal Scientific Center of the East Asia Terrestrial Biodiversity, Far Eastern Branch of the Russian Academy of Sciences, 159, 100-Letia Vladivostoka Prospect, 690022 Vladivostok, Russia; gontcharov@biosoil.ru (A.A.G.); artyrozz@mail.ru (A.Y.N.); nikulinvyacheslav@gmail.com (V.Y.N.); 3 MUSE—Museo delle Scienze, Limnology & Phycology Section, Corso del Lavoro e della Scienza 3, I-38123 Trento, Italy

**Keywords:** charophytes, Egypt, aquatic habitats in oases, multifaceted approach, North Africa, phylogenetics, rare species, desert springs

## Abstract

Present-day information available on the charophyte macroalgae in Egypt, including their phylogenetic affinities, remains largely incomplete. In this study, nine charophyte populations were collected from different aquatic biotopes across the Egyptian Western-Desert Oases and Sinai Peninsula. All populations were investigated using an integrative polyphasic approach including phylogenetic analyses inferred from the chloroplast-encoded gene (*rbc*L) and the internal transcribed spacer (ITS1) regions, in parallel with morphotaxonomic assignment, ultrastructure of the oospore walls, and autecology. The specimens identified belonged to the genera *Chara*, *Nitella*, and *Tolypella*, with predominance of the first genus to which five species were assigned though they presented some interesting aberrant taxonomic features: *C. aspera*, *C. contraria*, *C. globata*, *C. tomentosa*, and *C. vulgaris*. Based on our integrative study, the globally rare species *C. globata* was reported for the second time for the whole African continent. The genus *Nitella* was only represented by *N. flagellifera*, and based on the available literature, it is a new record for North Africa. Noteworthy, an interesting *Tolypella* sp., morphologically very similar to *T. glomerata*, was collected and characterized and finally designated with the working name ‘*Tolypella* sp. PBA–1704 from a desert, freshwater wetland’, mainly based on its concatenated *rbc*L+ITS1 phylogenetic position. This study not only improved our understanding on the diversity, biogeography and autecological preferences of charophytes in Egypt, but it also broadened our knowledge on this vulnerable algal group in North Africa, emphasizing the need of more in-depth research work in the future, particularly in the less–impacted desert habitats.

## 1. Introduction

Charophytes (Charales, Streptophyta), including both extant and fossil members of the order Charales (besides members of the extinct orders Sycidiales and Moellerinales), constitute an ancient group of terrestrial autotrophic macroalgae, the ancestors of which invaded land and developed to the present-day land plants 450 million years ago [1,2,3]. Ecologically, members of the family Characeae are widely distributed in freshwater and brackish biomes [4,5,6,7], with rare occurrence in marine habitats [8,9]. They play a keystone role in maintaining the balance and functioning of the ecosystems they colonize. Therefore, a better understanding of the ecological preferences of this vulnerable algal group is important for the conservation and restoration of their habitats [10,11]. Charophytes are known to be highly vulnerable to water pollution and eutrophication, and they therefore are one of the most severely threatened groups of algae [11,12,13]. Their sensitivity to different ranges of water pollution, in particular nutrient enrichment, allows to use them effectively as excellent proxies for environmental assessments [14,15,16]. Fossil charophyte gyrogonites have also been used as a complementary tool for paleolimnological reconstruction, particularly in arid and hyper-arid regions in North Africa [17,18,19,20]. To accurately delineate the species identity of members of the family Characeae, the application of integrative polyphasic approaches, combining molecular phylogenetic data, morphotaxonomic traits and ecology, has nowadays become an important prerequisite [21,22,23,24,25], particularly if unusual taxonomic characters are present and reflecting peculiar phenotypic adaptations to their natural ecosystems [26,27,28,29].

In North Africa, particularly in the Maghreb countries and starting from the late 19th century, the family Characeae attracted the attention of many researchers, and hence a wealth of information is available on this group of algae from the morphological and ecological standpoints for this geographic area [5,15,30,31,32,33], and the references therein]. Corillion [32] reported 54 species and intraspecific taxa. Several years later, Muller et al. [5] reviewed the charophytes of Morocco, Algeria, and Tunisia, based on examination of herbarium specimens and freshly sampled materials, and revealed 31 morphospecies. Some regionally limited species, such as *Chara strigosa* A.Braun and *C. tomentosa* L., have also been reported. Their study also included some Mediterranean endemic and endangered species, such as *C. imperfecta* A.Braun, *C. oedophylla* G.Feldmann (currently accepted taxonomically as *C. vulgaris* var. *oedophylla* (G.Feldmann) R.D.Wood), and *C. vulgaris* var. *gymnophylla* (A.Braun) A.Braun, besides the typical tropical taxa *C. zeylanica* Klein ex Willdenow and *Lamprothamnium succinctum* (A.Braun) R.D.Wood. In their study on the charophytes inhabiting wetlands of Numidia in north-eastern Algeria, Zouaïdia et al. [15] reported *Chara braunii* C.C.Gmelin, *C. galioides* A.P.De Candolle, *Nitella gracilis* (J.E.Smith) C.Agardh, and *N. hyalina* (De Candolle) C.Agardh as rare species, as well as *Nitella batrachosperma* C.Agardh (currently accepted taxonomically as *N. confervacea* (Brébisson) A.Braun ex Leonhardi), found in a very-clean water pool as a new record for Algeria. All the above-mentioned taxa posed conservation values for their habitats and are currently designated as rare species in the Maghreb countries [4,5,15], confirming the need of more intensive surveying and in-depth taxonomic studies on this endangered algal group in North Africa, particularly in the face of the ongoing global climatic changes and land-use impacts.

In Egypt, knowledge about the diversity and ecological preferences of the streptophytes, including the stoneworts, is still grossly limited [34,35]. The initial contribution on charophyte diversity is due to Braun [30] whilst the last five decades generally saw a progress in the understanding of their biodiversity and distribution. Corillion and Guerlesquin [36] and Corillion [32] identified 26 charophyte species, with 24 taxa assigned to the genus *Chara* and only one species each for the genera *Nitella* (*N. opaca*) and *Tolypella* (*T. nidifica*). They emphasized the necessity of more intensive surveying studies on the family Characeae in Egypt to have a complete picture on this group of algae and also advocated the need of taxonomic revision for certain species such as *Chara diaphana* (F.J.F.Meyen) R.D.Wood. Several years later, a few studies reported some cosmopolitan taxa sporadically, mainly from different desert biotopes [37,38,39,40,41]. All these studies were based only on morphotaxonomic analyses and limited ecological data. Over the last five years, multifaceted studies on the Egyptian stonewort flora, based on freshly-sampled and herbarium specimens and including a combination of molecular, morphological, and ecological data, were initiated [42,43,44].

The goal of this study was to identify morphotaxonomic diagnostic traits, phylogenetic affinities, and autecological preferences of charophyte populations collected from different Egyptian biotopes in the Western-Desert Oases and Sinai Peninsula to improve our current limited understanding on the biogeography and diversity of charophytes in Egypt and, generally, in North Africa.

## 2. Results and Discussion

### 2.1. Phylogenetic Affinities of the Charophyte Specimens Investigated

**Figure 1:**
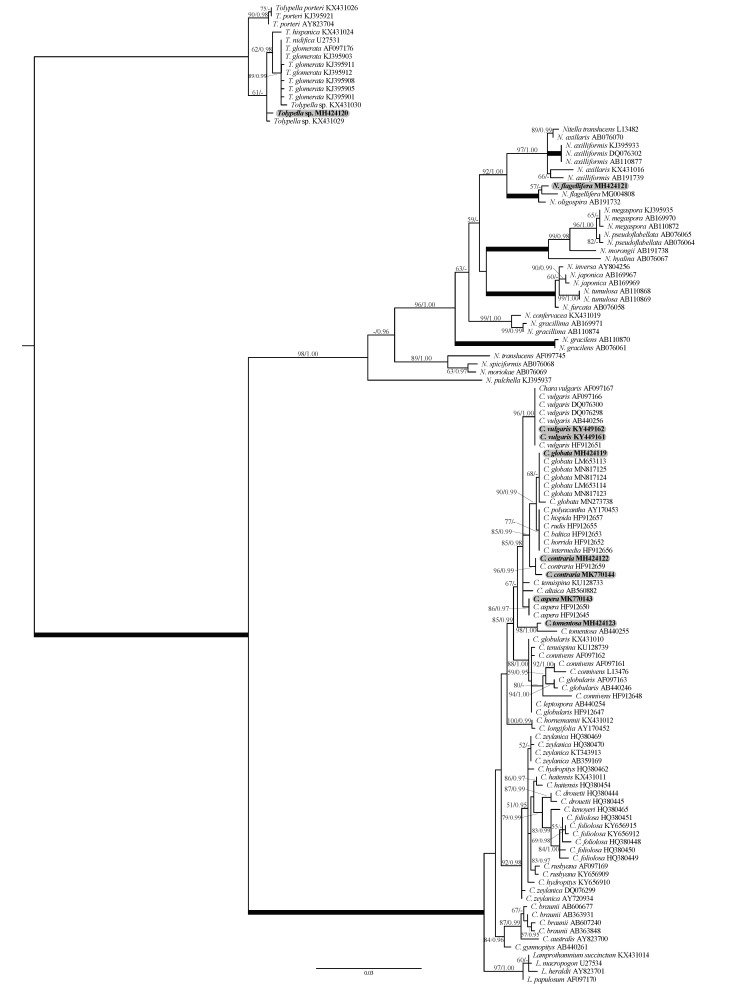
Phylogeny of Characeae based on 121 *rbc*L sequences of *Chara*, *Nitella*, *Tolypella*, and *Lamprothamnium*. ML tree was inferred in PAUP with GTR+I+G nucleotide substitution model with maximum likelihood bootstrap values (>50%) and posterior probabilities (>0.95) shown at branches. Branches received 100% BP and 1.00 PP support, and the newly obtained sequences are shown in bold.

**Figure 2:**
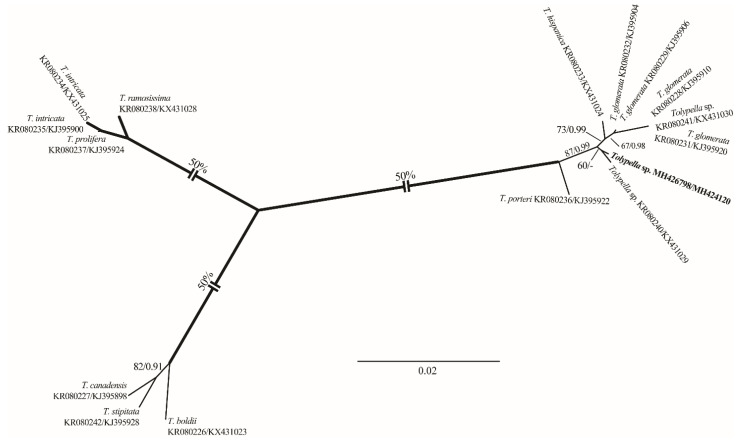
Phylogeny of *Tolypella* based on 16 concatenated *rbc*L and ITS1 sequences. ML tree was inferred in PAUP with TrN+G nucleotide substitution model. See the legend of Figure 1 for details. Long internal branches were graphically reduced 50%.

**Figure 3:**
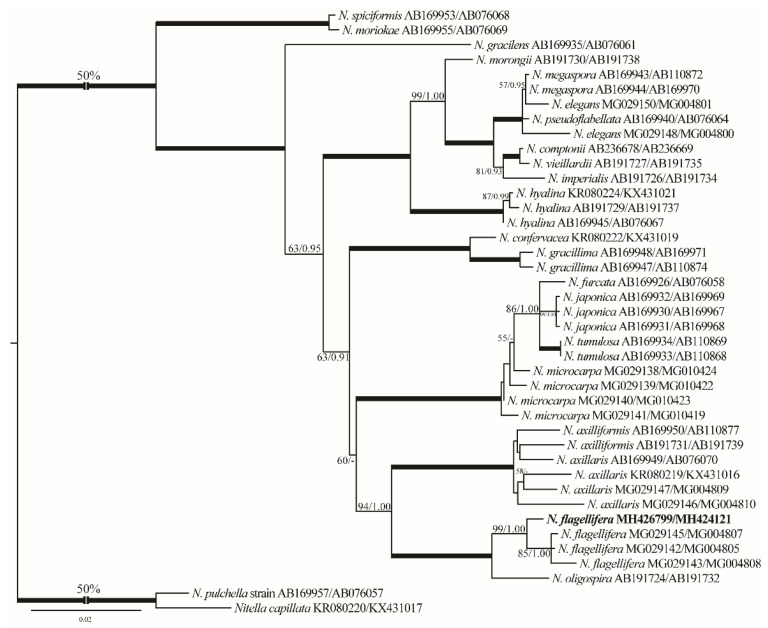
ML-phylogenetic tree inferred in PAUP with GTR+I+G nucleotide substitution model using 41 concatenated *rbc*L and ITS1 sequences of *Nitella*. See the legend of Figure 1 for details.

**Figure 4:**
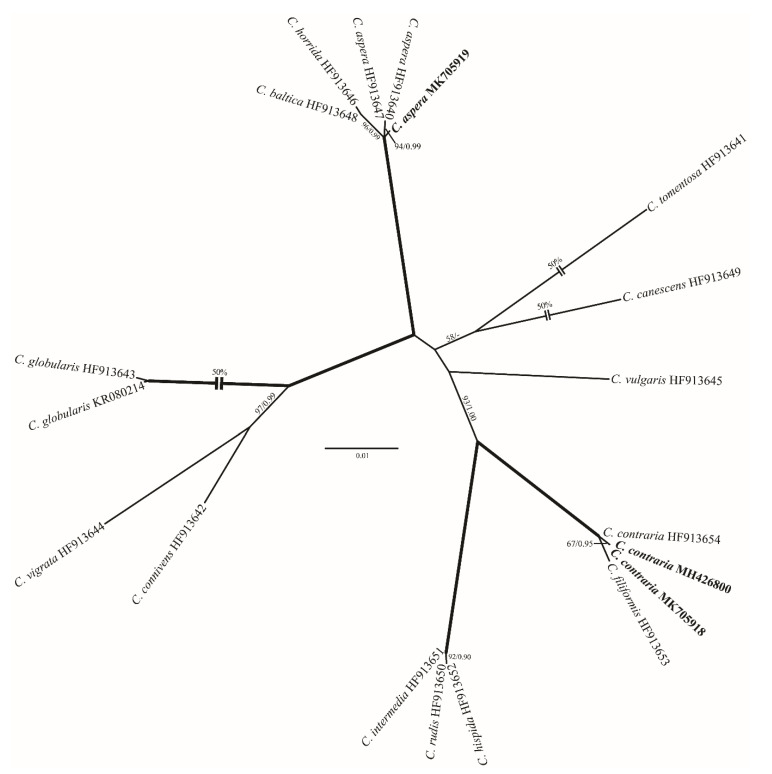
Phylogeny of *Chara* (19 accessions) based on ITS rDNA sequence comparisons. The tree was inferred in PAUP with HKY+G nucleotide substitution model. See the legend of Figure 1 for details.

To aid morphology-based identification process we assembled a dataset of 121 *rbc*L sequences of charophytes representing major genera (Table S1). The alignment included 70 *Chara* accessions, 32 *Nitella* sequences, 15 *Tolypella* sequences, and 4 *Lamprothamnium* species. Representatives of these genera formed robust (*Tolypella*) or strongly supported (*Nitella* and *Lamprothamnium*) generic clades. *Chara* was resolved only topologically as a sister of *Lamprothamnium* (98/1.00; Figure 1). All our *Chara* sequences were assigned to well-supported species clades (*C. vulgaris*, *C. globata*, *C. contraria*, *C. aspera*, and *C. tomentosa*). Similarly, in the genus *Nitella* the new sequence was placed in the robust *N. flagellifera* clade. Only our *Tolypella* accession occupied unresolved position in a weakly supported clade. In the analyses with the concatenated data set that included 16 *rbc*L and ITS1 sequences of *Tolypella*, our sequence showed weak affinity to *Tolypella* sp. from Australia (Figure 2). Combined chloroplast and nuclear markers provided additional support for many internal clades in the genera *Nitella* (Figure 3) and *Chara* (Figure 4), and also confirmed affinities of *N. flagellifera*, *C. aspera*, and *C. contraria*.

### 2.2. Morphotaxonomy, Autecology, and Biogeography of the Charophyte Specimens Studied

In the present study, seven taxa belonging to the genera *Chara* (*C. aspera*, *C. contraria*, *C. globata*, *C. tomentosa*, and *C. vulgaris*), *Nitella* (*N. flagellifera*), and *Tolypella* (*Tolypella* sp. PBA–1704) were identified and discussed from the standpoints of morphotaxonomy and ecological characterization. The worldwide rare species *C. globata* is herein reported for the second time in the whole African continent. Interestingly, *N. flagellifera* represents the first record for both Egypt and North Africa. An interesting *Tolypella* sp., morphologically similar to *T. glomerata*, is designated with the working name ‘*Tolypella* sp. PBA–1704 from a desert, freshwater wetland’, mainly based on its concatenated *rbc*L+ITS1 phylogenetic position. Detailed descriptions, ecological preferences, and biogeography of all these taxa are given in the following. Hydrochemical characteristics of the habitats studied are provided in Table 1.

#### 2.2.1. *Chara aspera* Willdenow (Figure 5A–K)

**Figure 5:**
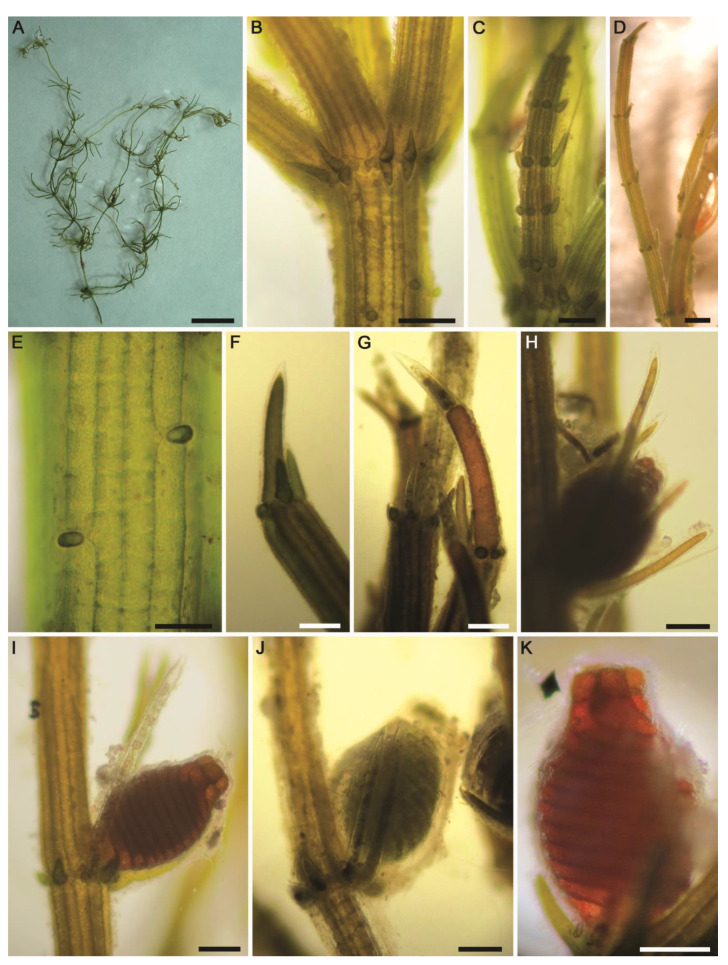
Morphotaxonomic features of *Chara aspera* found in a mineral spring-fed agricultural ditch in the Siwa Oasis (the Western Desert of Egypt): (**A**) Macroscopic habitus of female plant; (**B**) axial node showing stipulodes, papilliform spine-cells, and triplostichous tylacanthous cortex; (**C**,**D**) branchlets of female plant; (**E**) Triplostichous isostichous cortex; (**F**,**G**) apices of branchlets with 1–2-celled end segments; (**H**–**K**) parts of the branchlets depicting oogonia, bract-cells, bracteoles and coronula. Scale bars: (**A**) = 2 cm; (**C**,**D**) = 1 mm; (**B**,**F**–**K**) = 200 μm; (**E**) = 100 μm.

- Description: Plants green, dioecious, up to 40 cm tall, without incrustations (Figure 5A). Axes moderately slender, 350–450 µm in diameter. Cortex triplostichous, isostichous to tylacanthous (Figure 5B,E). Spine-cells solitary, papilliform (Figure 5B,E). Stipulodes diplostephanous (in 2 tiers), 2 sets per branchlet, acuminate, uppers somewhat longer than lowers (Figure 5B). Internodes corticated, 1–3 times longer than the branchlets (Figure 5A,B). Branchlets 6–9 in a whorl, straight and spreading, 1.2–1.8 cm long (Figure 5A); each branchlet consisting of 5–6 corticated segments (Figure 5C,D); end segment 1–2-celled, naked (Figure 5F,G). Bract-cells usually 5, well developed, unilateral, shorter to longer than oogonium (Figure 5H). Bracteoles 2, somewhat longer than the bract-cells and exceeding the mature oogonium (Figure 5I,J). Gametangia on separate plants and the female thalli only observed. Oogonia solitary at the 2–3 lowest branchlet nodes, 690–750 µm long (without coronula) × 450–500 μm wide, with 12–13 convolutions. Coronula 50–85 µm long × 50–100 (–120) μm wide (Figure 5K). Oospores and bulbils not observed.
- Distribution in Egypt: This charophyte species has already been recorded in Egypt [32].
- General distribution and ecology: Cosmopolitan species in Europe [4], Atlantic Islands [45], North America [46], Africa and Middle East [15,32,47], and Asia [48]. So far, it has not yet been recorded in South America, the Pacific Islands, and Australia [47]. In North Africa, Muller et al. [5] pointed out that this species is frequently common in coastal ponds and marshes. During the present study, the *Chara aspera* population was found in a mineral spring-fed agricultural ditch in the Siwa Oasis. Hydrochemical conditions in this Saharan biotope were as follows: high water temperature (°C): 27.7; neutral pH: 7.32; high electrical conductivity (μS·cm^−1^): 6350; Na^+^ and Ca^2+^ were the major cations: 1140 and 276 mg·L^−1^, respectively; Cl^−^ was the major anion: 2104 mg·L^−1^; low N, NO_3_^−^ and NH_4_^+^: 460 and 68 µg·L^−1^, respectively; high P, TP and SRP: 260 and 55 µg·L^−1^, respectively (Table 1). In a similar study, Zouaïdia et al. [15] reported *C. aspera* in nitrate-rich brackish wetlands (NO_3_^−^: 400–840 μg·L^−1^), with moderate orthophosphate levels (30–48 μg·L^−1^). Additionally, Caisová and Gąbka [49] and Urbaniak and Gąbka [50] highlighted that *C. aspera* has a wide range of ecological preferences in fresh and brackish calcareous waters, mainly in drainage canals and lakes.
- Remarks: There is general agreement between the characteristics of our *C. aspera* population and the information provided by Wood and Imahori [47]; however, the following aberrant taxonomic features were noted: (1) the spine cells are solitary and papilliform (vs. well-developed spine cells that may be solitary and in fascicles of 2–3 cells, often with bulbous bases, and up to 2.5 times as long as the axis diameter), (2) stipulodes are distinctly much shorter (vs. stipulodes 0.5–2 times as long as the axis diameter and often as long as the basal branchlet segment), (3) branchlets 6–9 in a whorl and each one consisting of 5–6 corticated segments (vs. 8–9 branchlets with 6–8 segments of which 5–7 are 2-corticate), (4) oogonia convolutions with 12–13 turns (vs. 13–15 turns in the protologue), and (5) coronula much smaller (i.e., 50–85 µm long × 50–100 (–120) μm wide vs. 75–100 µm long × 120–200 μm wide). In agreement with our taxonomic observations, the recent integrative study by Langangen et al. [51] on the charophytes inhabiting the warm Troll Springs in Svalbard (Spitsbergen) recorded a morphologically aberrant *C. aspera* population in these groundwater-dependent biomes, but the plants were still genetically identical to other specimens of *C. aspera* from several European countries. Taxonomically, they showed that these plants were ecorticated and sterile, stipulodes were absent, branchlets with 5–10 segments, cortex of the branchlets rudimentary or missing, and eventually branchlets tipped with 2–3 ecorticated cells. Our observations and findings of Langangen et al. [51] confirm the phenotypic variations in the spine-cells, stipulodes, and cortication in *C. aspera*. Accordingly, Blindow et al. [28] pointed out that the variability in lengths of spines, stipulodes, and bract cells, as well as branchlet cortication, are traits of limited value for species delineation.

#### 2.2.2. *Chara contraria* A.Braun ex Kützing (Figure 6A–K and Figure 7A–K)

**Figure 6:**
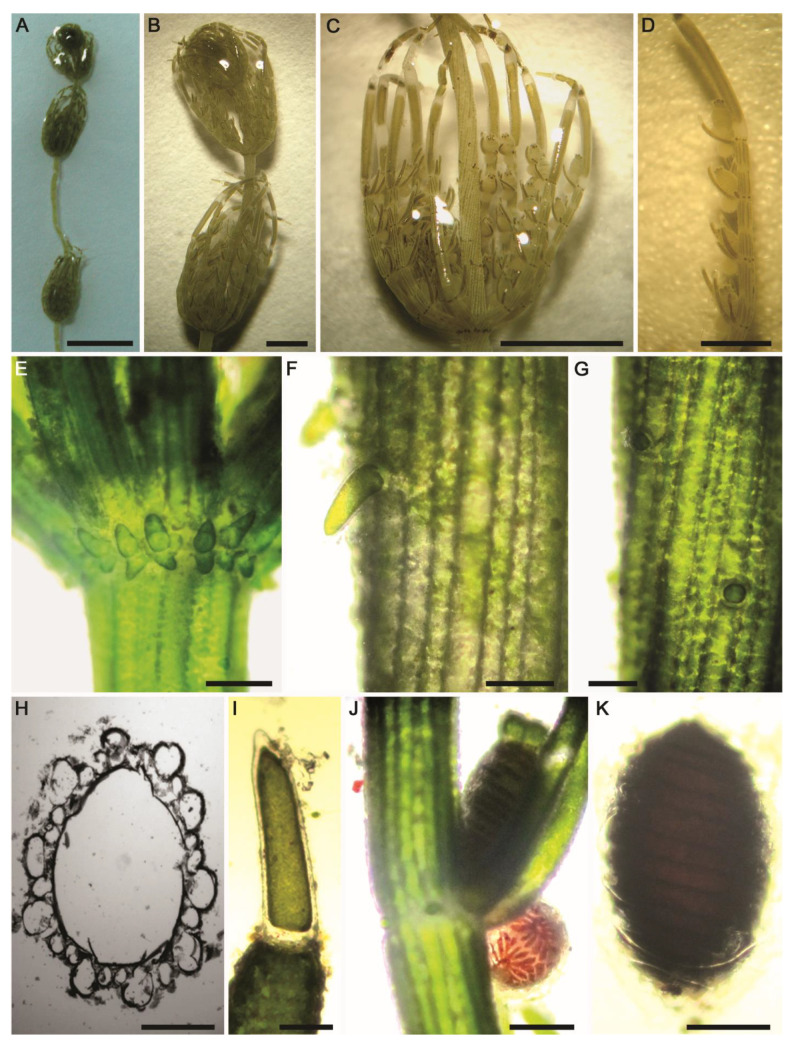
Morphotaxonomic features of *Chara contraria* found in a nutrient-rich artificial pool in the mountain valley “Wadi El-Arbaeen”, Saint Catherine Protectorate (South Sinai, Egypt): (**A**,**B**) macroscopic habitus; (**C**) whorl of branchlets; (**D**) branchlet; (**E**) axial node showing stipulodes; (**F**,**G**) stem cortex and spine-cells; (**H**) internode cross-section showing diplo–triplostichous cortex; (**I**) terminal branchlet cell; (**J**) node of branchlet depicting oogonia, bract-cells, and antheridia; (**K**) oospore. Scale bars: (**A**) = 2 cm; (**B**,**C**) = 0.5 cm; (**D**) = 2 mm; (**E**,**J**,**K**) = 200 μm; (**F**,**G**,**I**) = 100 μm; (**H**) = 50 μm.

**Figure 7:**
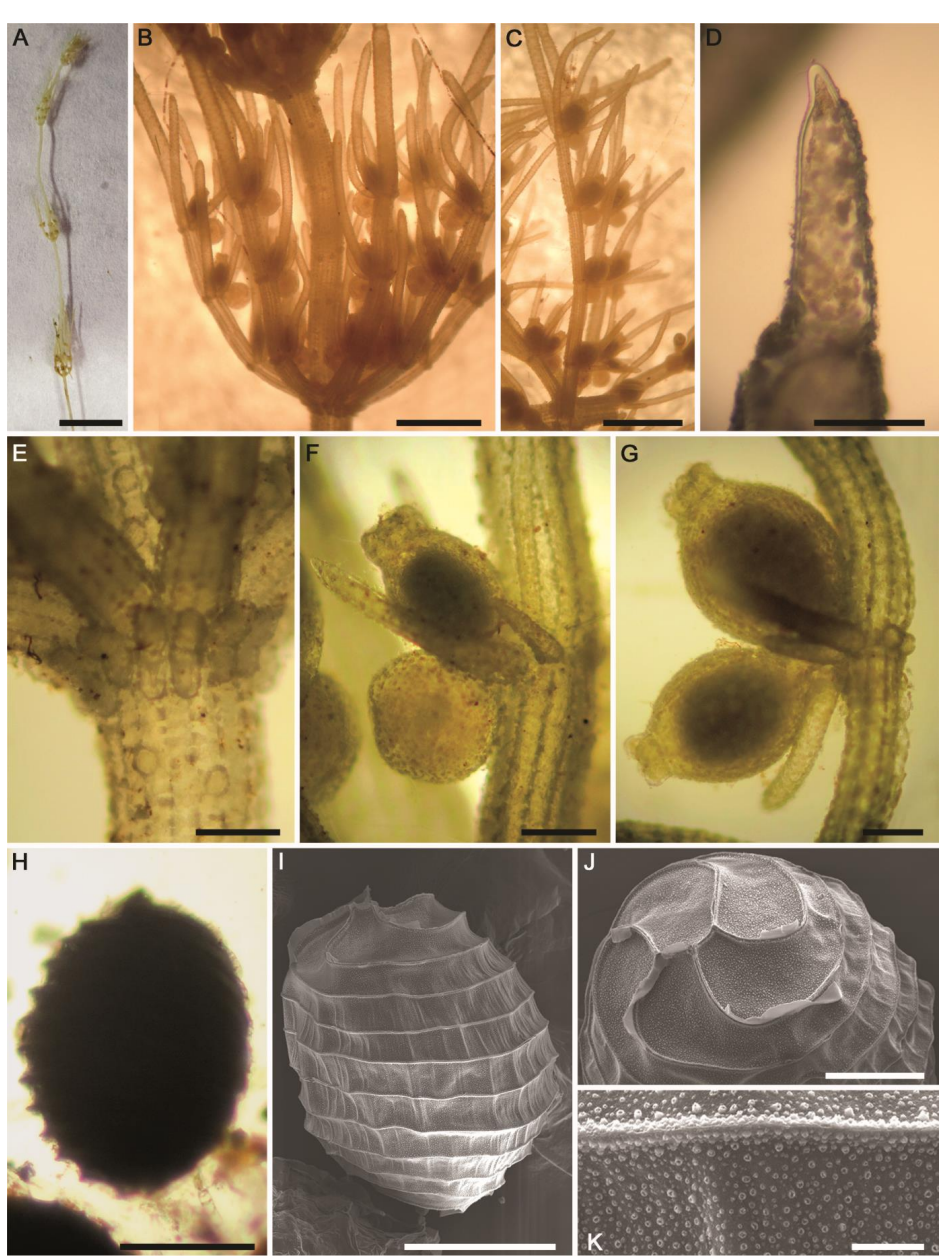
Morphotaxonomic features of *Chara contraria* found in an agricultural ditch in the El-Dakhla Oasis (the Western Desert of Egypt): (**A**) macroscopic habitus; (**B**) whorl of branchlets; (**C**) branchlet; (**D**) terminal branchlet cell; (**E**) axial node showing stipulodes and spine-cells; (**F**,**G**) nodes of branchlets showing oogonia and antheridia. Oogonia might be geminate. (**H**) Oospore; (**I**) SEM of the oospore; (**J**) apical part of the oospore, SEM; (**K**) close-up SEM view on the oospore wall showing granulate ornamentation covering the fossa and the ridges. Scale bars: (**A**) = 1 cm; (**B**,**C**) = 2 mm; (**E**–**I**) = 200 μm; (**D**,**J**) = 100 μm; (**K**) = 10 μm.

- Description: Plants olive green to green, monoecious, 15–50 cm tall, without incrustations (Figure 6A) or moderately incrusted (Figure 7A). Axes moderately slender, 350–685 µm in diameter. Cortex diplo- to triplostichous, isostichous to tylacanthous (Figure 6F–H and Figure 7E). Spine-cells variable, solitary, papilliform (Figure 6G and Figure 7E) or often shortly cylindrical (Figure 6F), up to 200 µm long. Stipulodes diplostephanous (in 2 tiers), 2 sets per branchlet, short, obtuse or blunt (Figure 6E and Figure 7E). Internodes corticated, 2–3 times as long as the branchlets, 3–4 cm long (Figure 6A and Figure 7A). Branchlets 7–10 in a whorl (Figure 6C and Figure 7B), incurved, (0.8–) 1–1.5 cm long; each branchlet consists of 5–7 segments of which the basal 3–5 segments corticated, diplostichous; end segment 2–3-celled, naked (Figure 6C,D and Figure 7C); terminal cell conical to long acuminate (Figure 6I and Figure 7D). Bract-cells 5, unilateral; anteriors longer than oogonium; posteriors smaller or rudimentary. Bracteoles 2, longer than the anterior bract-cells and 2–3 (–4) times longer than the mature oogonium (Figure 7C). Gametangia conjoined at each 1–4th branchlet nodes, solitary or rarely geminate, without mucus (Figure 6D and Figure 7C). Oogonia solitary (Figure 6J and Figure 7F) or geminate (Figure 7G), 590–825 µm long (without coronula) × 390–530 μm wide, with 13–14 convolutions. Coronula 90–100 (–110) µm long × 80–90 (–100) μm wide, cells oblong, blunt. Oospores dark brown to black (Figure 6K and Figure 7H), (490–) 670–710 µm long × 350–450 μm wide; striae of 10–14 prominent ridges (Figure 6K and Figure 7I), with rounded-shaped granulate ornamentation covering fossae and ridges (Figure 7J,K); fossae ca. 40–42 μm across. Antheridia small, 300–355 μm in diameter (Figure 6J and Figure 7F).
- Distribution in Egypt: Previously recorded in Upper Egypt [36].
- General distribution and ecology: Nearly cosmopolitan in all continents [4,47]. In North Africa, Muller et al. [5] showed that *Chara contraria* is rather rare in the Mediterranean region and can be found in various biotopes. With respect to its conservation status, Langangen [52] classified it as “Near Threatened” species. During the present study, it was found in a nutrient-rich artificial muddy pool in Wadi El-Arbaeen, Saint Catherine Protectorate, South Sinai and in an agricultural ditch in the El-Dakhla Oasis (the Western Desert of Egypt). Values of environmental variables were as follows: water temperature (°C): 20.6–21.9; circumneutral pH: 6.93–7.46; conductivity (μS·cm^−1^): 760–2960; Na^+^ and Ca^2+^ were the major cations: 62–361 and 62–147 mg·L^−1^, respectively; Cl^−^ was the major anion: 146–768 mg·L^−1^; low N, NO_3_^−^: 2320–5400 µg·L^−1^; NH_4_^+^: 57–924 µg·L^−1^; high P, TP and SRP: 150–1680 and 40–276 µg·L^−1^, respectively (Table 1). Thus, *C. contraria* can be found in circumneutral to slightly alkaline, fresh to slightly-brackish calcium-rich, meso–eutrophic waters. These observations are in agreement with the findings of Caisová and Gąbka [49] and Muller et al. [5].
- Remarks: Our specimens are consistent with the diagnosis of the protologue illustrated in Wood and Imahori [47,53]. Besides the clear-cut differences in some morphotaxonomic features with the most morphologically close species *C. vulgaris* (in particular tylacanthous cortication in *C. contraria vs.* aulacanthous in *C. vulgaris*), the two species are also well separated genetically (Figure 1).

#### 2.2.3. *Chara globata* W.Migula (Figure 8A–K, Figure 9A–K and Figure S3A–L)

**Figure 8:**
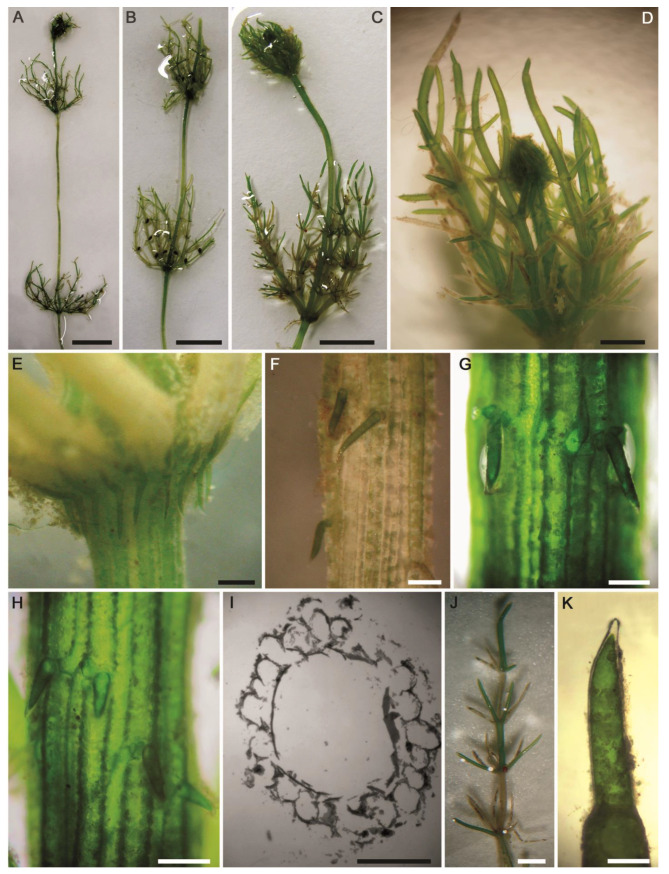
Morphotaxonomic features of *Chara globata* found in the thermal mineral desert spring ‘Ain Wazedi’ in the Siwa Oasis (the Western Desert of Egypt): (**A**–**C**) macroscopic habitus; (**D**) whorl of branchlets; (**E**) axial node showing stipulodes in 2 tiers; (**F**–**H**) axial cortex, diplo–triplostichous (slightly isostichous to distinctly tylacanthous), and spine-cells; (**I**) internode cross-section showing diplo–triplostichous cortication; (**J**) branchlet; (**K**) apex of branchlet. Scale bars: (**A**,**B**) = 1 cm; (**C**) = 0.5 cm; (**D**,**J**) = 2 mm; (**E**–**H**) = 400 μm; (**K**) = 200 μm, (**I**) = 40 μm.

**Figure 9:**
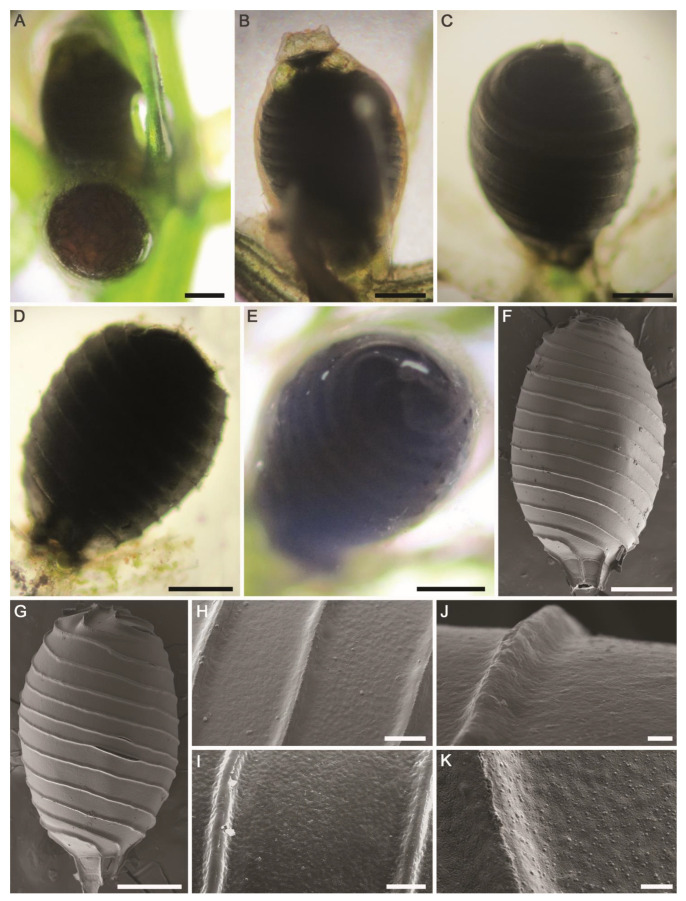
Morphotaxonomic features of *Chara globata* found in the thermal mineral desert spring ‘Ain Wazedi’ in the Siwa Oasis (the Western Desert of Egypt): (**A**) branchlet node with conjoined gametangia.; (**B**) fertilized oogonium; (**C**–**E**) mature oospores; (**F**–**K**) SEM of the oospores. Note smooth and pustular (to weakly papillate) ornamentation covering fossa and ridges. Scale bars: (**A**–**G**) = 200 μm; (**H**,**I**) = 20 μm; (**J**,**K**) = 5 μm.

- Description: Plants green to olive green (Figure 8A,B), monoecious, 20–85 (–95) cm tall, unencrusted to heavily incrusted, forming a massive growth inside the main springhead and the outlet channel of thermal mineral desert spring (Figure S3A,B). Axes predominately stout, 610–1580 (–2000) µm in diameter (Figure 8C and Figure S3C). The internodes usually longer than the branchlets, 1.5–4 (–5) times longer than the branchlets, up to 8 cm long (Figure 8A–C and Figure S3C), the upper parts of thalli look like spherical loose heads (Figure 8C,D). Cortex irregularly diplo- to triplostichous, slightly isostichous to distinctly tylacanthous (Figure 8F–I and Figure S3F). Spine-cells mostly solitary (Figure 8F,H and Figure S3E) or rarely in a bunch of four (only one very long and the other surrounding three distinctly very short) (Figure 8G), subulate, with thickened cell walls at their ends. Stipulodes diplostephanous (in 2 tiers), 2 sets per branchlet, well developed, long aculeiform with acute ends (Figure 8E and Figure S3D). The branchlets usually straight, but still slightly arcuate, 9–10 in a whorl, 1.5–2 (–2.5) cm long (Figure 8A–D and Figure S3C); each branchlet consists of 6–7 segments of which the basal 3–4 segments corticated, diplostichous; the distal segments 3–4 ecorticate (Figure 8D,J and Figure S3G); terminal cell distinctly acuminate and shorter than adjacent bract-cells (Figure 8K). Bract-cells 5–6, verticillate, strongly developed, acuminate, (1.5–) 3–4 mm in length (Figure 8D,J and Figure S3G). Bracteoles 2, usually shorter than the bract-cells. Gametangia conjoined, solitary (Figure 9A), occurring at the 3 lowest nodes between corticated segments (Figure 8C,J and Figure S3G). Oogonia solitary, 825–950 μm long (without coronula) × 535–590 μm wide, with 11–14 convolutions (Figure 9B). Coronula 100–120 µm long × 100–150 μm wide, cells more or less apiculate at apex (Figure 9B). Ripe oospores black (Figure 9C–E and Figure S3H), with 11–15 striae having prominent ridges and ending at the base with a basket–like protrusion (Figure 9F,G), 860–910 µm long (incl. protrusions) × 415–535 μm wide, oospore wall smooth (Figure 9H,J and Figure S3K) to pustular (Figure 9I, Figure S3L) to slightly papillate covering fossae and ridges (Figure 9K); fossae 50–87 μm across (Figure 9H,I). Antheridia solitary, octoscutate, 460–520 μm in diameter (Figure 9A).
- Distribution in Egypt: Only recorded in the Sinai Peninsula—the Asian part of the Egyptian territory—based on the recent study carried out by Romanov et al. [43] on the herbarium specimens collected from the end of the 1960s to the beginning of the 1970s from Sinai and stored at Tel Aviv University Herbarium.
- General distribution and ecology: Rare, but still flagship, temperate species with disjunctive biogeographical distribution, particularly in the arid and semiarid regions. Fresh–brackish, moderately alkaliphilic (pH: 7.1–8.0) species preferring waters rich in sodium sulphates, and calcium/magnesium bicarbonates [43]. So far, it has only been recorded in Asia (China, Iran, Israel, Kazakhstan, Kyrgyzstan, Russia, Turkmenistan and Uzbekistan) [43,47,54,55,56,57], Europe (Romania and the European part of Russia) [57,58], the Sahara–Arabian Desert in Sinai Peninsula [43], and in North Africa (only in Tunisia) [57]. During the present study, *C. globata* was found in the thermal mineral desert spring ‘Ain Wazedi’ in the Siwa Oasis. This Saharan biotope was characterized by the following hydrochemical characteristics:—high water temperature (°C): 26.5; circumneutral pH: 6.85; high conductivity (μS·cm^−1^): 6280; low DO (mg·L^−1^): 3.4; Na^+^ and Ca^2+^ were the major cations: 1430 and 94 mg·L^−1^, respectively; Cl^−^ and SO_4_^2−^ were the major anions: 1795 and 989 mg·L^−1^, respectively; low N, NO_3_^−^, NO_2_^−^ and NH_4_^+^: 200, 120, and 270 µg·L^−1^, respectively; high P, TP and SRP: 135 and 100 µg·L^−1^, respectively (Table 1).
- Remarks: The diagnostic taxonomic features of the Siwa *C. globata* population fitted better the specimens recently described by Romanov et al. [43] than the protologue redescribed by Wood and Imahori [47,53]. However, our specimens still differ from the description in Romanov et al. [43] by the following taxonomic features: (1) stem cortex irregularly diplo- to triplostichous, slightly isostichous to distinctly tylacanthous (vs. consistently a tylacanthous diplostichous stem cortex), (2) spine-cells mostly solitary, long acuminate and rarely in a bunch of four (only one very long and the other surrounding three distinctly very short) (vs. only solitary and variable in length from short conical–papillose to conical to long subulate), (3) gametangia usually present at the 3 lowest nodes of the corticated segments (vs. gametangia occurring at the 2–4 lowest nodes between corticated segments and rarely between ecorticate segments), and (4) ripe oospores are obviously dominant (vs. oospores low or absent in the majority of the specimens). We think that all these phenotypic variations are environmentally-induced and with a low taxonomic value. Taxonomically, Romanov et al. [43] also proposed that *C. globata* should be transferred and assigned to the subsection *Chara* in the section *Chara*, instead of the section *Grovesia* having a triplostichous stem cortex, in terms of the taxonomic observations obtained (i.e., consistently and generally tylacanthous diplostichous stem cortex, solitary spine–cells, and stipulodes in two tiers), corresponding well to the section *Chara* [47]. On the contrary, the Siwa *C. globata* specimens investigated in the present study are mainly characterized by the presence of isostichous to tylacanthous diplo- to triplostichous stem cortex (Figure 8F–I). Ling et al. [55] also documented irregular triplostichous tylacanthous cortex in Chinese specimens of *C. globata*. Additionally, the subsection *Chara* placement proposed by Romanov et al. [43] was not supported by crossing experiments conducted by Proctor [59,60], who pointed to the affinity of *C. globata* towards the subsection *Hartmania*. However, the combined morphotaxonomic and phylogenetic data obtained in this study (Figure 1), as well as work of Romanov et al. [43], showed that *C. globata* has more or less a closer affinity to species of the subsection *Hartmania* but that it is still different genetically and taxonomically (in particular in the presence of the verticillate bract-cells and arcuate branchlets mainly in the apical parts of thalli). In our opinion, the accurate taxonomic placement of *C. globata* is still problematic and more integrative studies are needed.

Our observations on the oospore wall ornamentation (mainly smooth to pustular, to slightly papillate, evenly covering fossa and ridges) coincide with the findings of Romanov et al. [43], and confirm one of the key diagnostic features for this rarely investigated species. In spite of *C. globata* having been recently recorded for the first time in North Africa in Tunisia, its morphotaxonomic diagnostic traits were poorly revealed (Figures 1c and 2e,f in [57]). In our polyphasic study, we are providing detailed information on the morphotaxonomy and on the phylogenetic affinity of the *C. globata* population in the Siwa Oasis, and these observations are novel for the whole African continent. Based on the *rbc*L phylogenetic analysis, *C. globata* is genetically distinct from the morphologically most allied taxa in the subsection *Hartmania*, such as *C. polyacantha*, *C. hispida*, *C. rudis*, *C. baltica*, *C. intermedia*, and *C. horrida*, and also placed separately within a clade that included only representatives of this geographically-limited species from Egypt and Israel (Figure 1). From the ecological standpoint, the Siwa Oasis *C. globata* population was found in the thermal mineral spring ‘Ain Wazedi’ and it can be considered as a flagship species in this unique biotope. This observation coincides with the findings of Romanov [57], who as well recorded this charophyte in an oasis-like locality in Tunisia. Spring habitats are well established as biodiversity hotspots, often also hosting rare and highly-specialized algal species [34,61]. It should also be recalled that Romanov et al. [43] recorded *C. globata* in the Sinai Peninsula, and emphasized rather little knowledge on the diversity of charophytes in the Sahara–Arabian Desert, indicating it as worthy of further studies. *C. globata* seems to be highly adapted and widely distributed in the Egyptian desert habitats (A.A.S. and co-workers, unpublished data), and it could therefore be considered as one of the characteristic *Chara* populations not only for Egypt but also for North Africa and the Sahara–Arabian Desert in general. We also think that the only available records of *C. hispida* var. *hispida* f. *polyacantha* (A.Braun) R.D.Wood and *C. hispida* var. *baltica* (Bruzelius) R.D.Wood from the Siwa Oasis [36] are misidentifications and indeed belong to *C. globata*. Although the morphotaxonomic traits of both taxa are not available in Corillion and Guerlesquin [36] for an in-depth check, they were sampled from the same oasis and share some morphological taxonomic features with *C. globata*. We predict that *C. globata* might be recorded in the future in the other Maghreb countries, particularly by applying combined morphological and phylogenetic approaches. Accordingly, it has been established that subtle species identification of members of the charophytes at the species and intraspecific level has nowadays become much easier thanks to the integrative polyphasic approaches, irrespective of the occurrence of populations showing marked phenotypic variability and developing so-called “phenoecodemes” as a result of the environmental and/or culture conditions [22,29,44].

#### 2.2.4. *Chara tomentosa* Linnaeus (Figure 10A–M)

**Figure 10:**
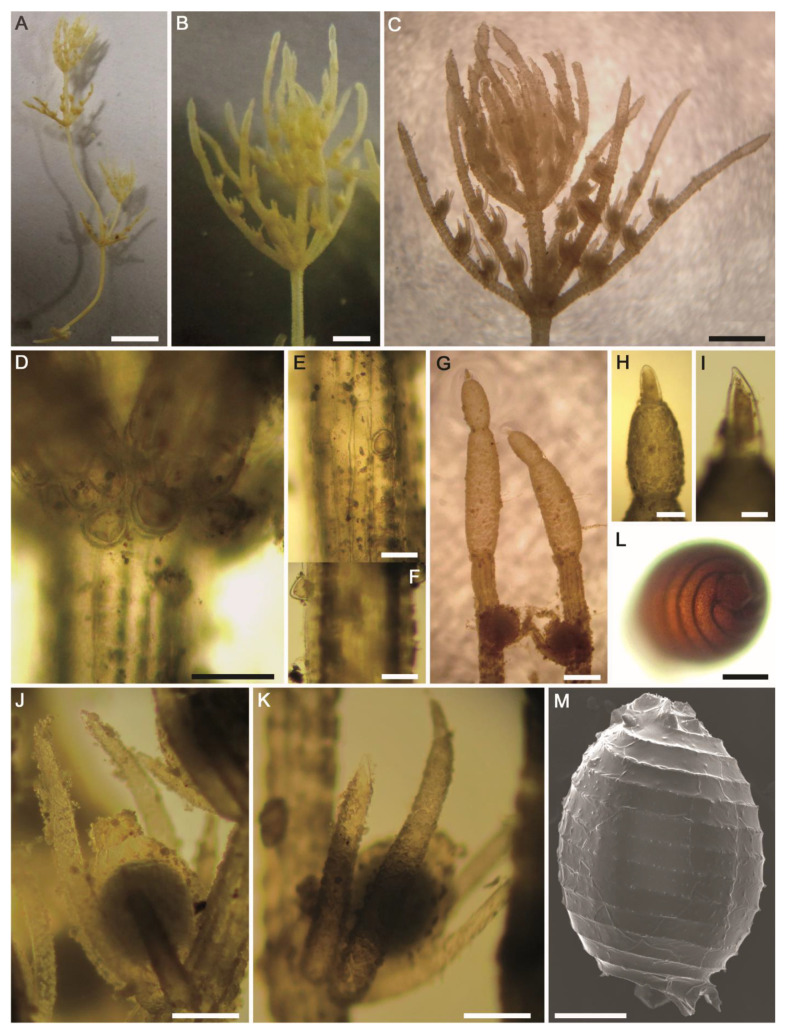
Morphotaxonomic features of *Chara tomentosa* found in a shallow marsh in the El-Dakhla Oasis (the Western Desert of Egypt): (**A**) macroscopic habitus; (**B**,**C**) whorls of branchlets of female plant; (**D**) axial node of young plant; (**E**,**F**) stem cortex and spine-cells; (**G**) branchlets of female plant with 2 inflated penultimate cells; (**H**,**I**) terminal branchlet cells; (**J**,**K**) nodes of branchlets with oogonia; (**L**) apical view of oospore; (**M**) SEM of oospore showing slightly flanged ridges, smooth ornamentation of fossae with irregularly small patterned projections, and basal claws. Scale bars: (**A**) = 1 cm; (**B**,**C**) = 2 mm; (**G**) = 500 μm; (**D**,**H**,**J**,**K**) = 200 μm; (**E**,**F**,**L**,**M**) = 100 μm; (**I**) = 50 μm.

- Description: Plants dioecious, small, robust, moderately to heavily encrusted, parts of the plants red in color, up to 8 cm tall (Figure 10A). Axes stout, 410–480 µm in diameter (Figure 10B). Stem cortex diplostichous, tylacanthous (Figure 10E). Internodes 1.5–2.0 (–2.5) times longer than the branchlets, upper internodes much shorter, up to 3 cm long (Figure 10A,B). Spine-cells variable, mostly solitary, sometimes ovoid with thickened walls (Figure 10E,F). Stipulodes diplostephanous (in 2 tiers), 2 sets per branchlet, short, ovoid (Figure 10D). Branchlets 6 in a whorl, slightly incurved, 1–1.2 cm long; each branchlet consisting of 6–7 segments of which the basal 4–5 segments 2-corticated (Figure 10C); end segment 2–3-celled, ecorticated; penultimate cells swollen, constricted at the nodes, broader than other segments (Figure 10G); terminal cell acute to mucronate (Figure 10H,I). Bract-cells 5, verticillate, acuminate; anteriors longer than oogonium; posteriors much smaller or similar to anteriors (Figure 10J). Bracteoles 2, somewhat longer than anterior bract-cells, acuminate (Figure 10K). Gametangia on separate plants, female thalli only observed. Oogonia solitary, heavily incrusted, at each 1st–3rd lowest branchlet nodes (Figure 10C), 450–490 µm long (without coronula) × 345–360 μm wide, mostly with 14 convolutions. Coronula 90–115 µm long × 95–125 μm wide. Oospores brown to dark brown (Figure 10L), 450–485 µm long × 295–317 μm wide; striae of 13–14 prominent and slightly flanged ridges apparently prolonged at the base into claws, with smooth to irregularly patterned small projections covering fossae and ridges; fossae ca. 30–33 μm across (Figure 10M).
- Distribution in Egypt: This charophyte species has been previously recorded in the Siwa Oasis [36].
- General distribution and ecology: Europe [4], North Africa [5,47], and Asia [62]. Old records in North and South Americas resulted from erroneous identifications [47]. Caisová and Gąbka [49] highlighted that it is occasionally present in marshes and large fish ponds. Urbaniak and Gabka [50] reported its occurrence in meso- to eutrophic calcium-rich lakes. In North Africa, it has been categorized as a fresh–brackish, rare species with a limited distribution in the Maghreb countries [5]. As regards its conservation status, Stewart and Church [63] considered it to be a “vulnerable” species in Britain and Ireland. During the present study, *C. tomentosa* was found in a brackish, calcium-rich, shallow marsh in the El-Dakhla Oasis. The main physical and chemical features of this Saharan biotope were: high temperature (°C): 27.2; slightly alkaline pH: 7.53; high conductivity (μS·cm^−1^): 7700; DO (mg·L^−1^): 7.1; Na^+^ and Ca^2+^ as major cations: 1072 and 654 mg·L^−1^, respectively; Cl^−^ and SO_4_^2−^ as major anions: 2543 and 582 mg·L^−1^, respectively; NO_3_^−^ and NH_4_^+^: 1765 and 114 µg·L^−1^, respectively; TP and SRP: 715 and 295 µg·L^−1^, respectively (Table 1).
- Remarks: The diagnostic taxonomic features of the *C. tomentosa* population in our study better fitted the description illustrated by Corillion and Guerlesquin [36] than the specimens redescribed by Wood and Imahori [47,53] in the following: (1) branchlet segments 6–7 of which the basal 4–5 segments corticated and end segment 2–3-celled, ecorticated vs. 3–5(–6) segments of which (1–)2–3(–5) corticated and 1–3 naked, and (2) oogonia present at each 1–3th lowest branchlet nodes vs. 2nd–3rd lowest branchlet nodes [47,53]. The smaller dimensions of oogonia and oospores might be related to the desert environmental conditions.

#### 2.2.5. *Chara vulgaris* Linnaeus (Figure 11A–O)

**Figure 11:**
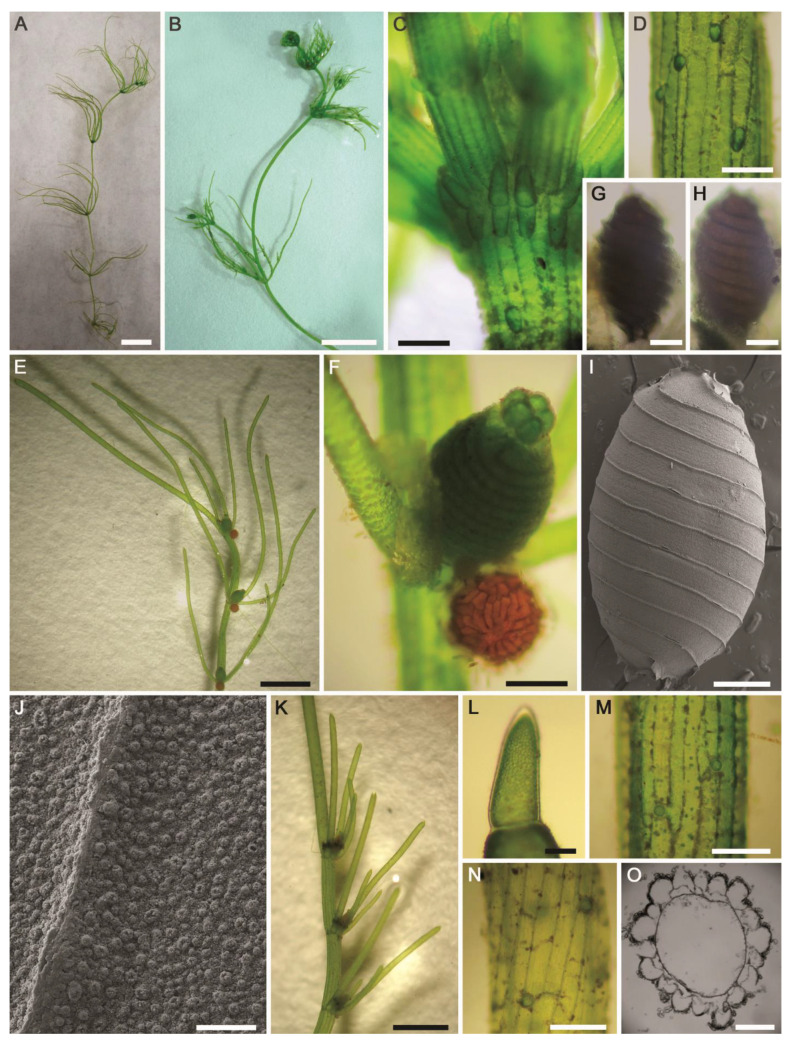
Morphotaxonomic features of *Chara vulgaris* found in the Siwa (**A**–**J**) and El-Farafra (**K**–**O**) Oases (the Western Desert of Egypt): (**A**,**B**) macroscopic habitus; (C) axial node showing stipulodes; (**D**) diplostichous aulacanthous cortex; (**E**) branchlet; (**F**) conjoined gametangia; (**G**–**I**) oospores; (**J**) SEM of oospore showing granulate ornamentation with pores on fossae and ridges; (**K**) branchlet; (**L**) terminal branchlet cell; (**M**,**N**) diplo- to slightly irregularly triplostichous cortex; (**O**) internode cross-section cross. Scale bars: (**A**,**B**) = 1 cm; (**E**,**K**) = 2 mm; (**C**,**D**,**F**,**M**,**N**) = 200 μm; (**G**,**H**,**I**) = 100 μm; (**L**,**O**) = 50 μm; (**J**) = 5 μm.

- Description: Plants monoecious, green in color, without incrustations, up to 45 cm tall (Figure 11A,B). Axes slender or moderately stout, 400–650 µm in diameter. Stem cortex diplostichous, generally aulacanthous (Figure 11D,O) to more or less isostichous (Figure 11C,M,N). Internodes 1–2 (–2.5) times as long as the branchlets, upper internodes much shorter, up to 3 cm long (Figure 11A,B). Spine-cells variable, solitary, papilliform (Figure 11M,N) or short, obtuse (Figure 11D). Stipulodes diplostephanous (in 2 tiers), 2 sets per branchlet, short, oblong-ovate (Figure 11C). Branchlets (6–)7–9(–10) in a whorl, incurved in the upper parts of the plants (Figure 11B) and more spreading in the lower parts of the plants (Figure 11A), 1–2 cm long; segments 4–5 of which the basal 3–4 segments 2-corticated, poorly developed; end segment 1–2-celled (Figure 11E,K), ecorticated; end cell conical (Figure 11L). Bract-cells unilateral, anteriors developed, 1–2 pairs longer than oogonium (about 5–13 times the length of oogonia), exceeding adjacent segment; posteriors usually rudimentary or absent (Figure 11E,K). Bracteoles similar to anterior elongated bract-cells. Gametangia conjoined at the 1–3th lowest branchlet nodes (adjacent to corticated segments) (Figure 11E,K). Oogonia solitary, 472–590 µm long (without coronula) × 300–415 μm wide, with 13–14 convolutions. Coronula 70–95 µm long × 55–74 μm wide (Figure 11F). Oospores dark brown to black, 495–565 µm long × 295–320 μm wide (Figure 11G,H); striae of 11–12 prominent and slightly flanged ridges (Figure 11I), with granulate ornamentation (with pores) on both fossae and ridges (Figure 11J); fossa ca. 45 μm across. Antheridia solitary, octoscutate, 260–390 in diameter (Figure 11F).
- Distribution in Egypt: Previously recorded in a pond at the barrage [36], Ain Radi in the Siwa Oasis [37], a freshwater streamlet near the Abu Hatab village, El-Sharkeia governorate [64], Holocene lacustrine sediments of Qarun Lake [18], and the thermal Springs of Moses, Sinai Peninsula [44].
- General distribution and ecology: Cosmopolitan species in all freshwater biotopes, and occasionally in brackish waters [4,49]. It typically occurs in meso–eutrophic habitats [15]. In North Africa, it is very common in the Maghreb countries [5]. During the present study, it was found in the outlet channel of the thermal mineral desert spring ‘Ain Al-Maamal’ in the Siwa Oasis, and an agricultural ditch in the El-Farafra Oasis. Ranges of the physical and chemical variables determined: water temperature (°C): 23.8–31.6; slightly-acidic to almost neutral pH: 6.16–6.71; high conductivity (μS·cm^−1^): 960–4470; Na^+^ and Ca^2+^ as major cations: 25–545 and 30–135 mg·L^−1^, respectively; Cl^−^ was the major anion: 131–1235 mg·L^−1^; SO_4_^2−^: 36–300 mg·L^−1^; HCO_3_^−^: 221–314 mg·L^−1^; NO_3_^−^: 200–400 µg·L^−1^; NH_4_^+^: 150–271 µg·L^−1^; TP and SRP: 20–100 and 17–48 µg·L^−1^, respectively (Table 1). In agreement with our ecological assessment, Zouaïdia et al. [15] pointed out that *C. vulgaris* is a species tolerating hyper-eutrophic water conditions (up to 780 μg·L^−1^ for PO_4_^3−^, 898 μg·L^−1^ for NO_3_^−^, and 140 μg·L^−1^ for NO_2_^−^).
- Remarks: Strong resemblance of our population with the one described by Wood and Imahori [47]. Although our specimens are characterized by the presence of elongated bract-cells, approximately 5–13 times the length of oogonia, they still genetically and taxonomically belong to the polymorphic species *Chara vulgaris*; not to the variety *longibracteata*. The same observation has already been reported by Saber et al. [44] during their integrative study on an aberrant *C. vulgaris* population from the Springs of Moses on the Sinai Peninsula, where they noticed that the bract-cells and bracteoles were clearly longer (ca. 4–12 times) than the oogonia. Similarly, Muller et al. [5] considered this phenotypic variation as a North-African “morphotype/phenoecodeme” due to high insolation.

#### 2.2.6. *Nitella flagellifera* J.Groves & G.O.Allen (Figure 12A–M)

**Figure 12:**
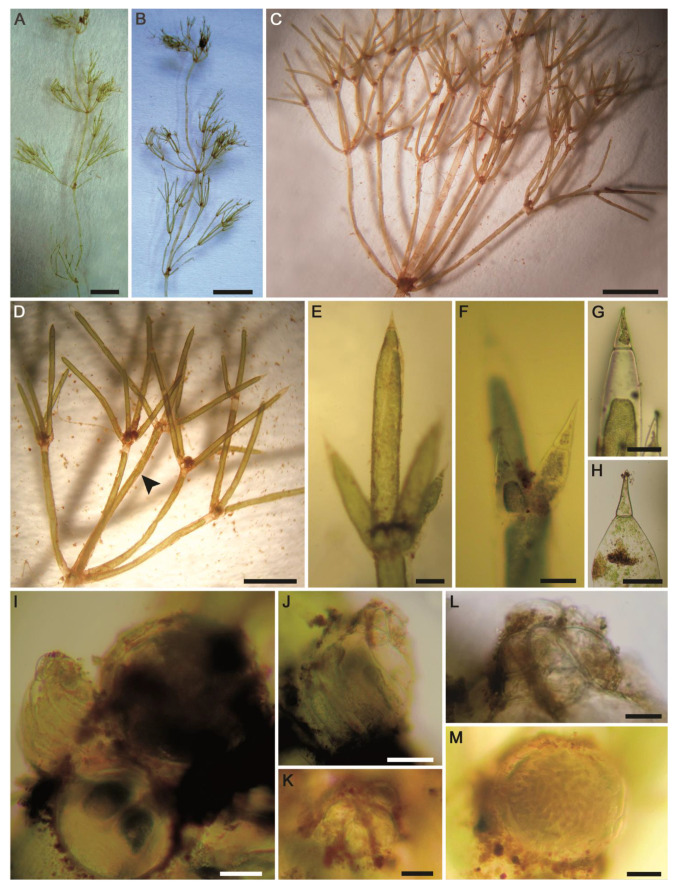
Morphotaxonomic features of *Nitella flagellifera* found in an agricultural ditch in the El-Dakhla Oasis (the Western Desert of Egypt): (**A**,**B**) macroscopic habitus; (**C**) 2–3-furcate branchlets; (**D**) fertile branchlet with gametangia at the third nodes. Note the central secondary ray (arrowhead); (**E**,**F**) dactyls; (**G**,**H**) end cells of dactyls; (**I**) conjoined gametangia; (**J**) oogonium; (**K**) apical view of coronula; (**L**) details of coronula; (**M**) antheridium. Scale bars: (**A**,**B**) = 1 cm; (**C**) = 2 mm; (**D**) = 1 mm; (**E**) = 200 μm; (**F**–**H**) = 100 μm; (**I**–**M**) = 50 μm.

- Description: Plants monoecious, diffuse, delicate, pale green in color, up to 18 cm tall. Axes moderately slender, 400–435 µm in diameter. Internodes 1–2 times as long as the branchlets (Figure 12A,B). Branchlets fertile and sterile similar, 6–7 in a whorl, 3 cm long, 2–3 furcate, primary ray about half of the branchlet length, secondaries 4–5 of which one is the central ray and relatively more stout than the laterals, tertiaries 3–4 of which one may be central, quaternaries 2–4 (Figure 12C,D). Dactyls 2–4, 2-celled, elongated (Figure 12E,F); penultimate cell narrowed abruptly at distal end; end cell persistent, acute and conical, 100–120 µm long × 43–50 μm wide (Figure 12G,H). Heads not formed. Gametangia conjoined at the 1st–3rd lowest branchlet nodes, without mucous (Figure 12C,D). Oogonia solitary, (190–)200–225 µm long (incl. coronula) × 102–130 μm wide, with 7–8 convolutions (Figure 12I,J); coronula 50–68 µm long × 65–70 μm wide, upper cells more or less longer than lowers (Figure 12K,L). Oospores not observed. Antheridia solitary, 195–220 μm in diameter (Figure 12M).
- Distribution in Egypt: This is the first record of this charophyte both in North Africa and in Egypt.
- General distribution and ecology: According to current knowledge, this charophyte appears to have a limited biogeographical distribution. It has been recorded in Brazil, in South America [25,65,66,67], and Japan, India, and Bangladesh in Asia [47,68]. During the present study, *N. flagellifera* was found in a nutrient-rich agricultural ditch in the El-Dakhla Oasis, the Western Desert of Egypt. Main physical and chemical characteristics of this biotope were: high water temperature (°C): 31.5; alkaline pH: 8.13; high conductivity (μS·cm^−1^): 6670; low DO (mg·L^−1^): 3.8; Na^+^, K^+^ and Ca^2+^ as major cations: 1368, 136 and 110 mg·L^−1^, respectively; Cl^−^ and SO_4_^2−^ as major anions: 2378 and 356 mg·L^−1^, respectively; NO_3_^−^ and NH_4_^+^: 1570 and 86 µg·L^−1^, respectively; TP and SRP: 2750 and 421 µg·L^−1^, respectively (Table 1). Based on the TP and SRP concentrations, *N. flagellifera* can be considered an eutraphentic species that can tolerate high levels of pollution.
- Remarks: This species is considered a new record for Egypt and also for North Africa based on the published literature ([5,32,36,47] and references therein). Our *N. flagellifera rbc*L and ITS1 gene sequences are also the first ones for North Africa. From the taxonomic and phylogenetic points of view, our *N. flagellifera* specimens coincide with the specimens redescribed by Wood and Imahori [47] and also with the findings of Borges and Necchi [25]. Noteworthy, gametangia in our study were noticed at the first node of the branchlet (Figure 12C), and this taxonomic observation has also been documented for the Brazilian *N. flagellifera* population investigated by Borges and Necchi [25], and other previous studies (e.g., [66,67]). Contrarily, Wood and Imahori [47] noted the lack of gametangia at this position. Blindow et al. [69] pointed out the presence of high phenotypic plasticity and some taxonomic discrepancies in the key characters of the Subfamily Nitelleae, which hamper species identification. *N. flagellifera* also resembles morphologically and phylogenetically the closest species *N. oligospira* [25,47]. However, *N. flagellifera* is still different taxonomically by having a secondary central ray (Figure 12C,D), a unique taxonomic feature that can be easily used to distinguish it from *N. oligospira*. These two species have more or less similar distribution patterns, are phylogenetically closely related, and also occupy a distinctive position in the genus tree (Figure 3).

#### 2.2.7. *Tolypella* sp. (A.Braun) A.Braun (Figure 13A–J)

**Figure 13:**
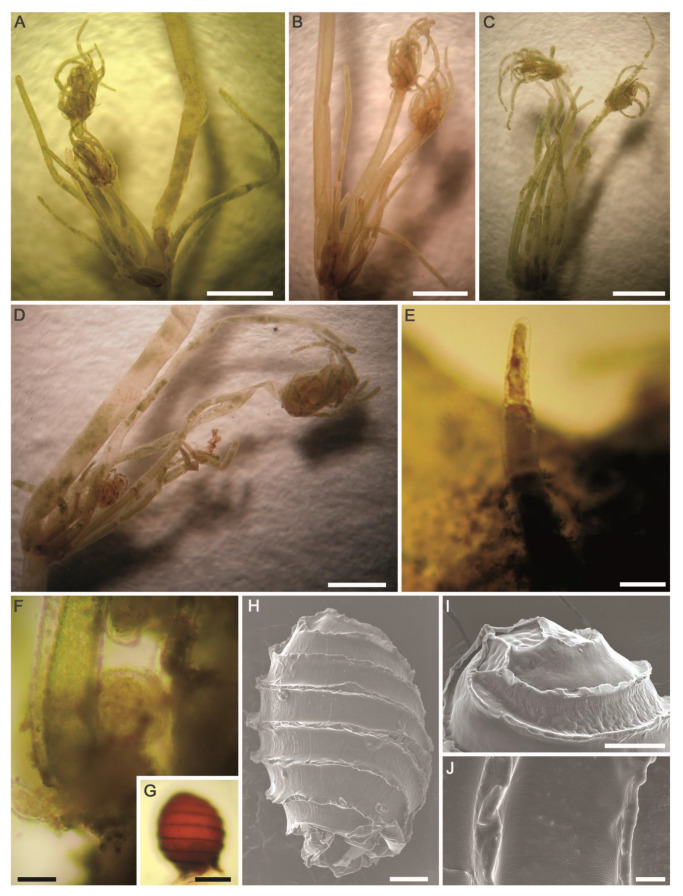
Morphotaxonomic features of *Tolypella* sp. PBA–1704 from a desert, freshwater wetland in the El-Dakhla Oasis (the Western Desert of Egypt): (**A**–**D**) macroscopic habitus; (**E**) fertile branchlet with cylindrical obtuse end cell; (**F**) branchlet node with gametangia; (**G**) oospore; (**H**) SEM of oospore showing flanged ridges; (**I**) apical view of oospore; (**J**) close-up SEM view on the oospore wall depicting smooth ornamentation of fossa and ridges. Scale bars: (**A**–**C**) = 3 mm; (**D**) = 2 mm; (**E**,**G**) = 100 μm; (**F**,**H**,**I**) = 50 μm; (**J**) = 10 μm.

- Description: Plants monoecious, pale green to green, unencrusted, fragile, up to 22 cm tall, with few coarse heads. Axes moderately slender, 500–850 µm in diameter. Internodes 1–2 times as long as the branchlets, becoming shorter towards the apex, up to 5 cm long. Sterile and fertile branchlets different (Figure 13A–D). The first node of the main axis produces 6–7 sterile branchlets and 2–4 secondary axes. The sterile branchlets are undivided and in a series of 3–5 elongated cells (Figure 13B,C). The fertile whorls produced by the secondary axes, short and grouped into fertile heads. Heads few to numerous, 3–14 per shoot (Figure 13A–D). The fertile branchlets apparently consist in a central row of cells (the “rachis”) that is a succession of nodes and internodes. These nodes carry the gametangia as well as 3 rays of 2–3 cells. All terminal cells are elongated, obtuse (Figure 13E). Gametangia conjoined at the fertile branchlet nodes, usually 1 central adaxial antheridium with 1–2(–3) lateral oogonia (Figure 13F). Oogonia 275–335 μm (incl. coronula) long × 250–280 μm wide, with 8–9 convolutions; coronula 30–35 μm high × 40–55 μm wide. Oospores brown to golden brown to slightly dark brown, (275–)320–354 μm long × 215–241 μm wide (Figure 13G); striae of 7–8 prominent, flanged ridges (Figure 13H,I); fossae and ridges with smooth ornamentation (Figure 13J); fossae 37–43 μm across. Antheridia solitary, small, sessile, 105–140 μm in diameter (Figure 13F).
- Distribution in Egypt: This is the first record worldwide of this genetically distinctive charophyte. We therefore designated it with the working name ‘*Tolypella* sp. PBA–1704 from a desert, freshwater wetland’ mainly based on its concatenated *rbc*L+ITS1 phylogenetic placement.
- General distribution and ecology: Our *Tolypella* sp. specimens only showed a weak affinity to *Tolypella* sp. from Australia based on the concatenated data set of 16 *rbc*L and ITS1 sequences. During the present study, *Tolypella* sp. PBA–1704 was found in a shallow meso–eutrophic wetland in the El-Dakhla Oasis, the Western Desert of Egypt. This biotope had the following hydrochemical characteristics: relatively-low temperature (°C): 19.2; slightly-alkaline pH: 7.64; medium-low conductivity (μS·cm^−1^): 360; low DO (mg·L^−1^): 3.3; Ca^2+^ as the major cation: 38 mg·L^−1^; SO_4_^2−^ and HCO_3_^−^ as major anions: 88 and 82 mg·L^−1^, respectively; NO_3_^−^ and NH_4_^+^: 421 and 19 µg·L^−1^, respectively; TP and SRP: 217 and 62 µg·L^−1^, respectively (Table 1).
- Remarks: In spite of the high morphotaxonomic similarities between our *Tolypella* specimens and the cosmopolitan species *T. glomerata* [47], it is apparently still distinct phylogenetically from that taxon (Figure 1 and Figure 2), and we therefore designated it with the working name ‘*Tolypella* sp. PBA–1704 from a desert, freshwater wetland’ mainly based on its concatenated *rbc*L+ITS1 phylogenetic placement. Further in-depth taxonomic and molecular studies on this interesting *Tolypella* taxon are necessary to propose it as a (morphologically) cryptic species new to science or to recognize it as belonging to a wide genetic variability of *T. glomerata*.

## 3. Materials and Methods

### 3.1. Charophyte Sampling, Processing, and Morphological Identification

During sampling campaigns conducted from October 2016 to May 2018 to unravel the hidden phycological diversity in the Egyptian Oases and other comparable habitats, the charophyte populations investigated in the present study were collected from different aquatic biotopes, including thermal mineral desert springs, agricultural ditches, and shallow wetlands in the Western Desert Oases (Siwa, El-Dakhla, and El-Farafra) and a nutrient-rich artificial muddy pool in the mountain valley “Wadi El-Arbaeen” in the Sinai Peninsula, Egypt (Table 2; Figures S1 and S2). The major water source in the Western Desert Oases is the Nubian Sandstone Aquifer System (NSAS), the world’s largest fossil freshwater reservoir [70]. In the Sinai Peninsula, only one population of *Chara contraria* could be sampled from Wadi El-Arbaeen. It is a mountain wadi located in the UNESCO world heritage site “Saint Catherine Protectorate”. The only water source in this mountain valley is the shallow aquifers that are typically recharged by heavy rainfalls [71]. Charophyte specimens were collected in clean sterile polyethylene terephthalate bottles and then transported to the laboratory where the specimens were cleaned with tap water to be carefully analyzed under the light microscope. A part of each specimen collected was also dried for the DNA extraction and sequencing. The specimens were identified following primarily Wood and Imahori [47,53] and Krause [4]. The key morphotaxonomic characters were checked and determined with the aid of a Novex^®^ RZT stereomicroscope (EUROMEX microscopes BV, Arnheim, the Netherlands), and a BEL^®^ photonics biological light microscope (BEL^®^ Engineering, Monza, Italy), and the light microscopy (LM) micrographs were taken with a Canon Powershot G12 digital camera. The biometric data provided were based on a minimum of 20–25 measurements for each character per species. The oospores were treated with acetic acid to remove any lime–shell, washed with distilled water and cleaned from the spiral cells by adding 10% Triton X100, and then stored at 60 °C for at least 10 h [43]. They were washed again with distilled water and sonicated to completely get rid of the spiral cells. The cleaned oospores were stored in 95% alcohol. To characterize detailed architecture of the oospore walls they were mounted, air-dried onto small round aluminum stubs, sputtered with chromium (Cr), and then studied with a Sigma^®^ 300 VP electron microscope (Carl Zeiss AG, Oberkochen, Germany) at 3.0–20.23 kV at the A.V. Zhirmunsky National Scientific Center of Marine Biology, Far Eastern Branch of the Russian Academy of Sciences, Vladivostok, Russia. The terminology used to describe the oospore surface follows Urbaniak [72]. All photos were digitally manipulated, and plates were created using Adobe Photoshop 8.0^®^ (Adobe Inc., California, USA). Voucher specimens were deposited in the collections of the Phycology Unit (No. 341) of the Botany Department, Faculty of Science, at Ain Shams University, Cairo (Egypt), curated by Abdullah A. Saber, under the accession numbers PBA–1801, PBA–1603, PBA–1701, PBA–1604, PBA–1702, PBA–1601, PBA–1602, PBA–1703, and PBA–1704 for *Chara aspera* from the Siwa oasis, *C. contraria* from Wadi El-Arbaeen (South Sinai), *C. contraria* from El-Dakhla Oasis, *C. globata* from the Siwa Oasis, *C. tomentosa* from El-Dakhla Oasis, *C. vulgaris* from the Siwa Oasis, *C. vulgaris* from El-Farafra Oasis, *Nitella flagellifera* from El-Dakhla Oasis, and *Tolypella* sp. from El-Dakhla Oasis, respectively.

### 3.2. Hydrochemical Characterization of the Sampling Sites

In situ water temperature, pH, ion conductivity, and total dissolved solids (TDS) were measured with a calibrated HANNA HI 991301 m (Hanna^®^ Instruments Co., Ltd., Woonsocket, RI, USA). Dissolved Oxygen (DO) was measured in the field with a Lutron^®^ YK-22DO (Lutron Electronic Enterprise Co., Ltd., Taipei City, Taiwan) oxygen meter. Hydrochemical analyses followed standard methods [73,74]. Anions (Cl^−^, CO_3_^2−^, HCO_3_^2−^, and SO_4_^2−^), major cations (Na^+^, K^+^, Ca^2+^, and Mg^2+^), and metals were estimated using ionic chromatography (ICS 1500 Dionex Corp.). Nutrients (NO_3_^−^, NO_2_^−^, NH_4_^+^, soluble reactive phosphorus SRP, and total phosphorus TP) were measured by molecular absorption spectrometry. Silicates (SiO_2_) were analyzed by the molybdosilicate method.

### 3.3. DNA Extraction, Amplification, and Sequencing

Total genomic DNA was extracted as described by Echt et al. [75] with some modifications [76]. Polymerase chain reaction (PCR) amplification was performed using the Encyclo Plus PCR kit (Evrogen, Moscow, Russia) with a T100 Thermal Cycler (Bio-Rad Laboratories, Hercules, CA, USA). The *rbc*L gene was amplified and sequenced in two fragments, using the following primer pairs for PCR: *rbc*L-RH1 [77] and *rbc*L-972R, for the 5′-gene fragment; and *rbc*L-295F [78] and *rbc*L-1379R ([79] with modifications) for the 3′-fragment. The PCR cycling profile included an initial step of 3 min at 95 °C, followed by 38 cycles of denaturation at 95 °C for 20 s, 20 s of annealing at 49 °C, and 1 min at 72 °C, with a final extension at 72 °C for 5 min. The ITS1 rDNA region was amplified using primers ITS-36F and ITS-IR [80]. The PCR cycling profile for this region included a denaturation at 95 °C for 3 min, followed by 38 cycles of denaturation at 95 °C for 20 s, annealing at 55 °C for 20 s, elongation at 72 °C for 1 min and a final extension step at 72 °C for 5 min. The PCR products were purified by ExoSAP-IT PCR Product Cleanup Reagent (Affymetrix, Santa Clara, CA, USA) and sequenced in both directions using an ABI 3500 genetic analyzer (Applied Biosystems, Foster City, CA, USA) with a BigDye terminator v3.1 sequencing kit (Life Technologies Corporation, Austin, TX, USA) and the same primers used for PCR. Sequences were assembled with the Staden Package v1.4 [81], aligned manually in the SeaView program [82]. The sequences were deposited in GenBank (Table 2).

### 3.4. Phylogenetics Analyses

Maximum likelihood (ML) analysis was carried out using PAUP 4.0b10 [83]. Bayesian inference (BI) was performed using MrBayes 3.1.2 [84]. To determine the most appropriate DNA substitution model for our datasets, the Akaike information criterion (AIC; [85]) was applied with jModelTest 2.1.1 (Table 3; [86]). ML analysis was done using heuristic searches with a branch-swapping algorithm (tree bisection-reconnection). Some parameters of ML and BI were listed in Table 3. In BI, convergence of the two chains was assessed, and stationarity was determined according to the ‘sump’ plot with the first 25% of samples discarded as burn-in; posterior probabilities were calculated from trees sampled during stationary phase.

The robustness of the ML trees was estimated by bootstrap percentages (BP; [87]) and posterior probabilities (PP) in BI. BP < 50% and PP < 0.95 were not taken into account. ML-based bootstrap analysis was inferred using the web service RAxML version 7.7.1 (http://embnet.vital-it.ch/raxml-bb/; accessed on 2 May 2021; [88]).

## 4. Conclusions

This study improved and updated our understanding on the taxonomic status, species diversity and autecological niches of nine charophyte populations colonizing different biotopes in the Egyptian Western-Desert Oases (North Africa) and Sinai Peninsula. *Nitella flagellifera* is here recorded for the first time in Egypt and North Africa.

An interesting *Tolypella* sp. has been designated with the working name ‘*Tolypella* sp. PBA–1704 from a desert, freshwater wetland’, based on its distinct position in *rbc*L+ITS1 placement from the morphologically similar *T. glomerata*. In spite of the fact that most *Chara* taxa we recorded are cosmopolitan and eurytopic [4,47], our integrative study confirmed the occurrence of the worldwide rare species *C. globata* for the second time in North Africa. The surveys carried out in the present study have also made it possible to provide further information for some species already reported from Egypt, such as *C. aspera*, *C. contraria*, *C. tomentosa*, and *C. vulgaris* [32,36,44]. It should be stressed that most of the localities from which the aforementioned taxa had been previously reported from Egypt during the 6th and 7th decades of the last century have nowadays been degraded and often disappeared as a result of the immense human-mediated pressures and of the lack of governmental legislation to conserve this severely threatened algal group. In agreement with our conclusion, the recent study by Mjelde et al. [11] on the charophytes in Myanmar highlighted that eutrophication and direct human pressures on the freshwater habitats are among the main factors reducing charophyte diversity. Blindow [12] pointed out that eutrophication can cause competition among submerged macrophytes, a case which is physiologically unfavorable to the vulnerable species of charophytes.

In accordance with the SRP-based trophic system proposed by Lambert-Servien et al. [14] for the charophytes, the charophyte habitats we studied can be classified as meso–eutrophic, and, rarely, hyper-eutrophic (see TP and SRP values in Table 1). Therefore, the charophyte species identified can be considered eurytopic and P-enrichment-tolerant species. The relationships between charophyte distributional patterns and environmental variables, in particular nutrients, have been discussed in several previous studies [15,16,89]. To broaden our knowledge on this vulnerable group of algae in Egypt and North Africa, further studies applying polyphasic approaches based on sampling campaigns, in particular from the remote and isolated desert environments and from moderately impacted urban habitats, are needed. Ultimately, characterizing the eco-physiological adaptive strategies of this streptophycean group of algae is of pivotal importance to fill knowledge gaps about the mechanisms of their acclimatization to their harsh environmental conditions.

## Figures and Tables

**Table 1:** Hydrochemical variables of the sampling sites where the charophyte specimens were collected.

Parameter	Unit	*Chara aspera*	*Chara* *contraria*		*Chara* *globata*	*Chara* *tomentosa*	*Chara* *vulgaris*		*Nitella* *flagellifera*	*Tolypella* sp.
		(PBA–1801)	(PBA–1603)	(PBA–1701)	(PBA–1604)	(PBA– 1702)	(PBA–1601)	(PBA–1602)	(PBA–1703)	(PBA–1704)
Temperature	°C	27.7	21.9	20.6	26.5	27.2	31.6	23.8	31.5	19.2
pH		7.32	6.93	7.46	6.85	7.53	6.71	6.16	8.13	7.64
Conductivity	µS·cm^−1^	6350	760	2960	6280	7700	4470	960	6670	360
T.D.S.	mg·L^−1^	3260	400	2010	3110	6160	2240	460	5335	244
DO	mg·L^−1^	2.6	4.2	6.3	3.4	7.1	2.2	1.9	3.8	3.3
Ca^2+^	mg·L^−1^	276.0	61.8	146.9	94	653.7	134.9	29.9	110	38.2
K^+^	mg·L^−1^	59.3	19.3	89.7	28.6	146.6	32.1	24.8	135.6	18.4
Mg^2+^	mg·L^−1^	104	16.1	52	46.4	72.9	79.2	16.6	104.9	5.7
Na^+^	mg·L^−1^	1139.6	62.1	360.9	1430.3	1072.3	545	25.5	1368.2	17.2
Cl^−^	mg·L^−1^	2104.2	146.4	768.5	1794.8	2543.2	1235.5	131	2378.1	15
SO_4_^2-^	mg·L^−1^	399.8	74.6	356.5	989	582.2	300	36	355.7	88.1
HCO_3_^−^	mg·L^−1^	350.7	117.1	31.2	86.3	316.1	220.6	314.4	161.2	81.8
CO_3_^2−^	mg·L^−1^	0.0	0.0	0.0	0.0	0.0	0.0	0.0	0.0	0.0
NO_2_^−^	µg·L^−1^	15	462	0	120	0	11	8	46	0
NO_3_^−^	µg·L^−1^	460	5400	2320	200	1765	200	400	1570	421
NH_4_^+^	µg·L^−1^	68	924	57	270	114	271	150	86	19
TP	µg·L^−1^	260	150	1680	135	715	20	100	2750	217
SRP	µg·L^−1^	55	40	276	100	295	17	48	421	62
SiO_2_	mg·L^−1^	9.3	15.2	4.8	0.33	7.9	7.7	7.1	6.2	1.5
Fe	µg·L^−1^	475	264	1500	430	5500	19	59	2100	175
Mn	µg·L^−1^	105	120	315	120	191	1.30	4	450	17
Cu	µg·L^−1^	17	15	81	30	130	0.3	0.36	134	52
Zn	µg·L^−1^	135	11	255	300	360	1.7	8	263	81

DO, dissolved oxygen; TP, total phosphorus; SRP, soluble reactive phosphorus.

**Table 2:** Meta-data associated with each charophyte species investigated in our study.

Species	Codes	Site Description	Collection Date	Latitude (N)	Longitude (E)	Elevation (m)	GenBank Accession Numbers	
							*rbc*L	ITS1
*Chara* *aspera*	PBA–1801	mineral spring-fed agricultural ditch in the Siwa Oasis	6 May 2018	29° 13′ 9″	25° 31′ 59″	−9	MK770143	MK705919
*Chara* *contraria*	PBA–1603	nutrient-rich artificial muddy pool in Wadi El-Arbaeen, Saint Catherine Protectorate, South Sinai	6 October 2016	28º 32′ 14.8″	33º 57′ 41.8″	1732	MK770144	MK705918
	PBA–1701	agricultural ditch in El-Dakhla Oasis	6 March 2017	25° 29′ 32.687″	29° 6′ 52.889″	126	MH424122	MH426800
*Chara* *globata*	PBA–1604	thermal mineral desert spring ‘Ain Wazedi’ in the Siwa Oasis	14 October 2016	29° 14′ 24.3″	25° 29′ 45.4″	−20	MH424119	–
*Chara* *tomentosa*	PBA–1702	shallow marsh in El-Dakhla Oasis	7 March 2017	25° 32′ 13.48559″	29° 3′ 13.7921″	112	MH424123	–
*Chara* *vulgaris*	PBA–1601	outlet channel of the thermal mineral desert spring ‘Ain Al-Maamal’ in the Siwa Oasis	14 October 2016	29° 12′ 21.4″	25° 31′ 52.3″	−9.9	KY449161	–
	PBA–1602	agricultural ditch in El-Farafra Oasis	16 October 2016	27° 03′ 26.1″	27° 57′ 47.4″	104	KY449162	–
*Nitella* *flagellifera*	PBA–1703	agricultural ditch in El-Dakhla Oasis	6 March 2017	25° 33′ 35.6623″	28° 59′ 4.83688″	107	MH424121	MH426799
*Tolypella* sp.	PBA–1704	shallow wetland in El-Dakhla Oasis	6 March 2017	25° 33′ 56.68333″	28° 56′ 42.04014″	92	MH424120	MH426798

**Table 3:** Datasets’ characteristics.

Dataset/Parameter	*Chara*	*Chara*+*Nitella*+*Tolypella*	*Nitella*	*Tolypella*
Marker	ITS1	*rbc*L	*rbc*L+ITS1	*rbc*L+ITS1
Number of sequences	19	121	41	16
Model	HKY+G	GTR+I+G	GTR+I+G	TrN+G
Number of runs/Markov chains for BI	4/4	4/4	4/4	4/4
Number of generations for BI	600,000	10,000,000	1,000,000	500,000

## Data Availability

Data is contained within the article.

## Supplementary Materials

The following are available online at https://www.mdpi.com/article/10.3390/plants10061157/s1. Figure S1: General views on sampling sites from which the different charophyte populations were collected: (**A**–**C**) *Chara aspera* population in a mineral spring-fed agricultural ditch in the Siwa Oasis; (**D**–**F**) *C. contraria* population found in a nutrient-rich pool in the mountain valley “Wadi El-Arbaeen”, Saint Catherine Protectorate, South Sinai; (**G**,**H**) an agricultural ditch in the El-Dakhla Oasis where the *C. contraria* population was sampled; (**I**–**L**) the thermal mineral desert spring ‘Ain Wazedi’ in the Siwa Oasis where the biogeographically limited species *C. globata* was sampled. This relatively stiff and heavily encrusted population formed a massive growth inside the springhead and its outlet channel. Figure S2: General views on the sampling localities in the present study: (**A**,**B**) a shallow marsh in the El-Dakhla Oasis where small female plants of *Chara tomentosa* were collected; (**C**–**E**) the thermal mineral desert spring ‘Ain Al-Maamal’ in the Siwa Oasis where a *C. vulagris* population was found in its main outlet channel; (**F**) an agricultural ditch in the El-Farafra Oasis harboring a *C. vulagris* population; (**G**,**H**) an agricultural ditch in the El-Dakhla Oasis where *Nitella flagellifera* was found; (**I**) an interesting *Tolypella* sp. PBA–1704 population intermingled with other aquatic plants in a shallow wetland in the El-Dakhla Oasis. Figure S3: Additional images and micrographs showing the key taxonomic characteristics of the biogeographically rare species *Chara globata*: (**A**,**B**) heavily incrusted thalli; (**C**) whorl of branchlets; (**D**) Stipulodes; (**E**) solitary acuminate spine-cell; (**F**) axis cross-section; (**G**) branchlet; (**H**) ripe oospore; (**I**–**L**) details and ultrastructure of the oospore walls. Scale bars: (**A**,**B**) = 2 cm; (**C**) = 0.5 cm; (**G**) = 2 mm; (**E**) = 400 μm; (**D**,**H**,**I**,**J**) = 200 μm, (**F**,**K**) = 40 μm; (**L**) = 10 μm. Table S1: Datasets of *rbc*L and ITS1 sequences of charophytes included in the present study. Sequence-alignment-data (4 FASTA files in zipped directory).
