# Supplementary figures and images for: Integrative Taxonomic, Ecological and Genotyping Study of Charophyte Populations from the Egyptian Western-Desert Oases and Sinai Peninsula
Source: Plants (Basel). 2021 Jun 7;10(6):1157. doi: 10.3390/plants10061157 (PMC8226818; doi:10.3390/plants10061157)

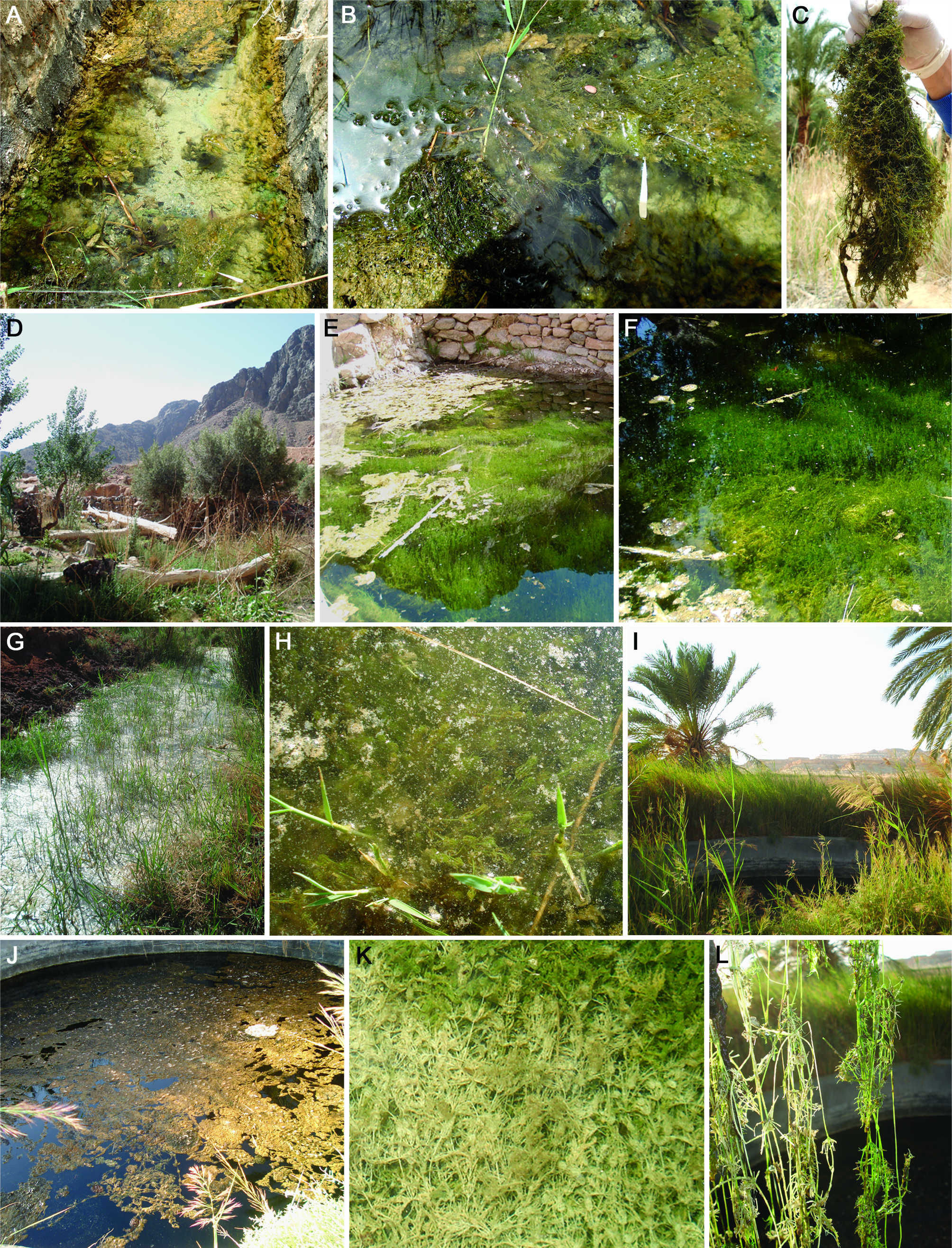

Supplement: Supplementary file 1 [file plants-10-01157-s001.zip › Supplemental-material_Egyptian-oases-charophytes/FigS1.tif]

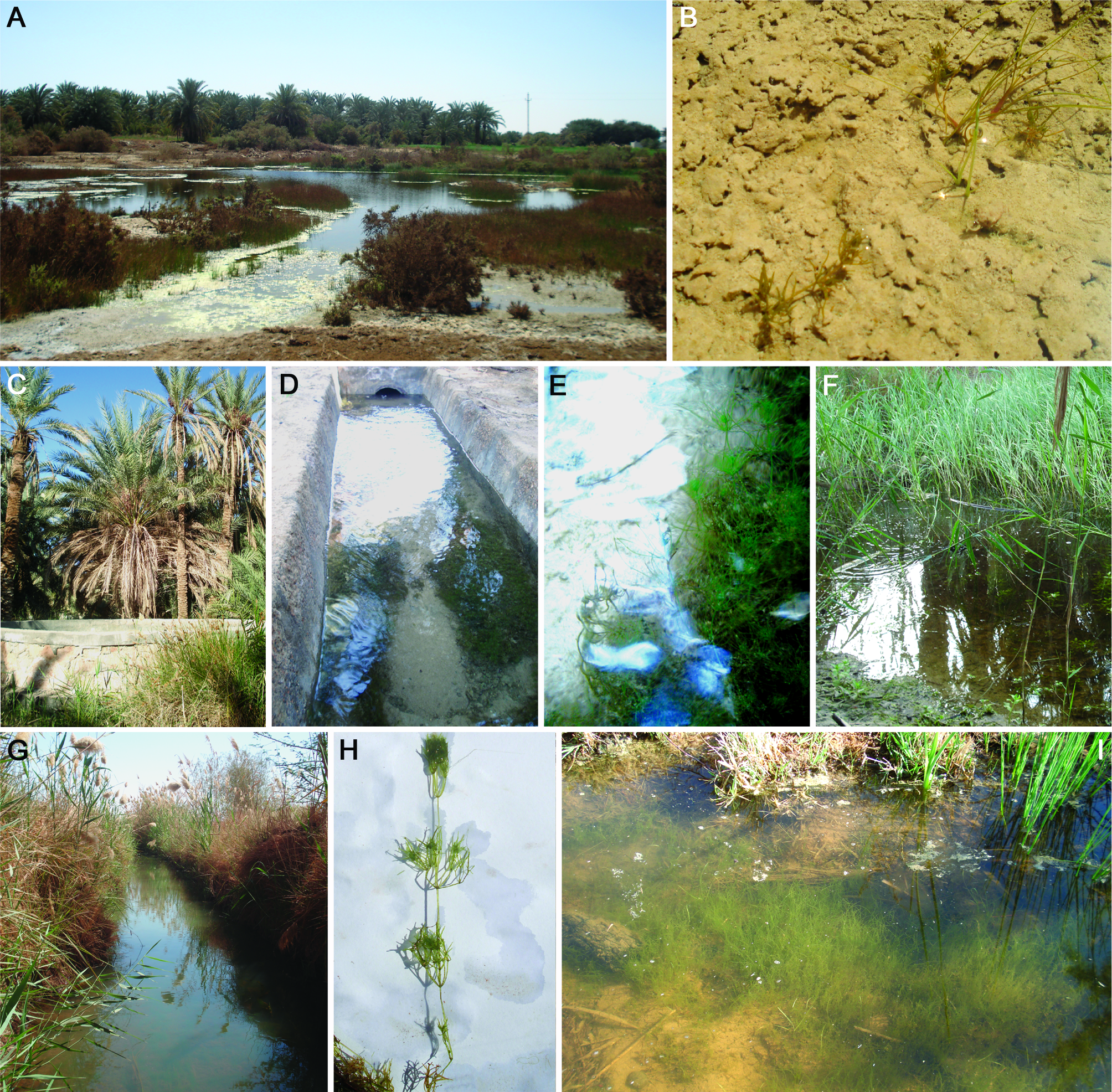

Supplement: Supplementary file 1 [file plants-10-01157-s001.zip › Supplemental-material_Egyptian-oases-charophytes/FigS2.tif]

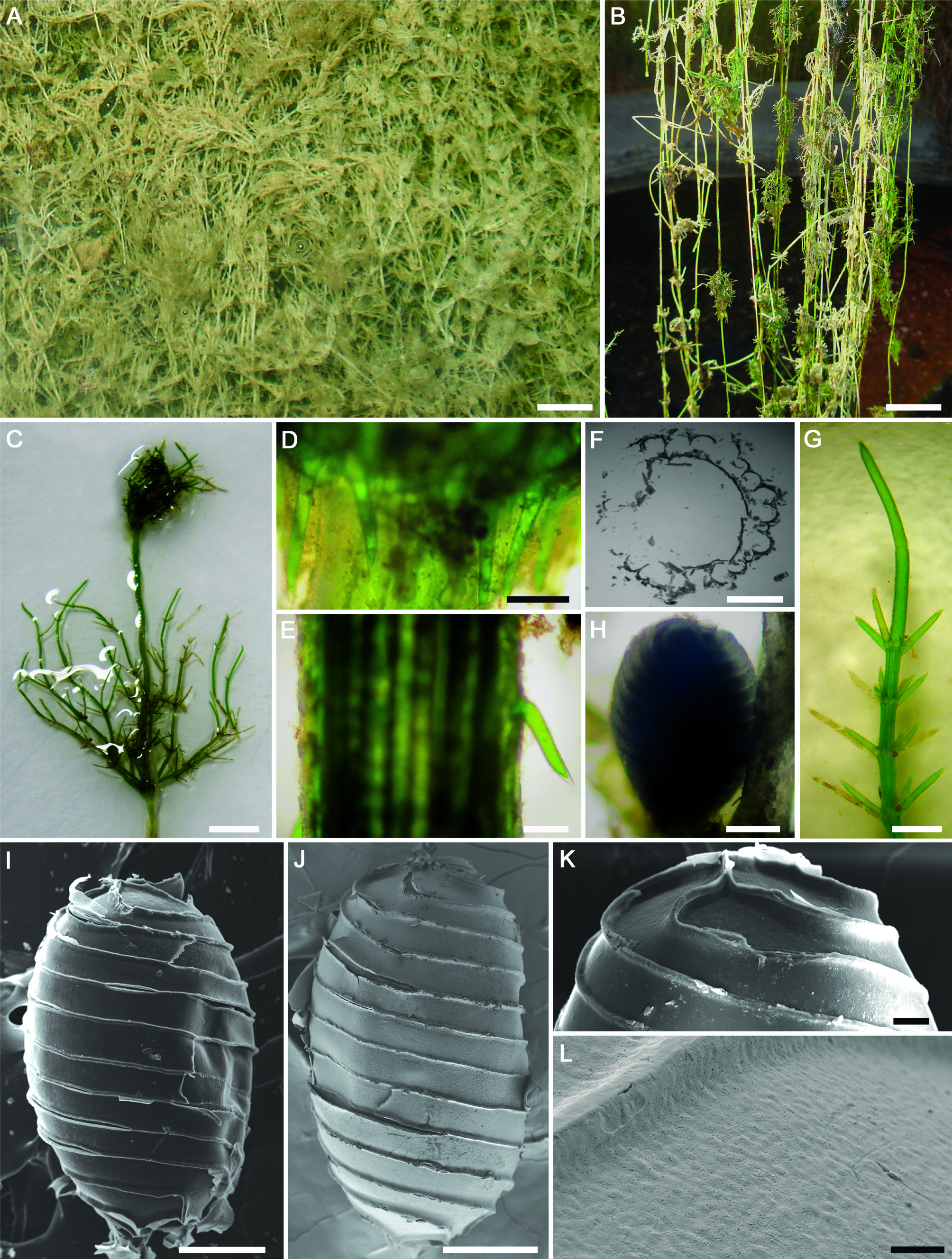

Supplement: Supplementary file 1 [file plants-10-01157-s001.zip › Supplemental-material_Egyptian-oases-charophytes/FigS3_Chara-globata.tif]
